# Metabolomic Analysis of Cold Acclimation of Arctic *Mesorhizobium* sp. Strain N_33_

**DOI:** 10.1371/journal.pone.0084801

**Published:** 2013-12-30

**Authors:** Abdollah Ghobakhlou, Serge Laberge, Hani Antoun, David S. Wishart, Jianguo Xia, Ramanarayan Krishnamurthy, Rupasri Mandal

**Affiliations:** 1 Soils and Crops Research and Development Centre, Agriculture and Agri-Food Canada, Quebec City, Quebec, Canada; 2 Department of Soils and Agri-Food Engineering, Laval University, Quebec City, Quebec, Canada; 3 Department of Biological Sciences, University of Alberta, Edmonton, Alberta, Canada; 4 Department of Computing Science, University of Alberta, Edmonton, Alberta, Canada; 5 National Research Council, National Institute for Nanotechnology (NINT), Edmonton, Alberta, Canada; Imperial College London, United Kingdom

## Abstract

Arctic *Mesorhizobium* sp. N_33_ isolated from nodules of *Oxytropis arctobia* in Canada’s eastern Arctic has a growth temperature range from 0°C to 30°C and is a well-known cold-adapted rhizobia. The key molecular mechanisms underlying cold adaptation in Arctic rhizobia remains totally unknown. Since the concentration and contents of metabolites are closely related to stress adaptation, we applied GC-MS and NMR to identify and quantify fatty acids and water soluble compounds possibly related to low temperature acclimation in strain N_33_. Bacterial cells were grown at three different growing temperatures (4°C, 10°C and 21°C). Cells from 21°C were also cold-exposed to 4°C for different times (2, 4, 8, 60 and 240 minutes). We identified that poly-unsaturated linoleic acids 18∶2 (9, 12) & 18∶2 (6, 9) were more abundant in cells growing at 4 or 10°C, than in cells cultivated at 21°C. The mono-unsaturated phospho/neutral fatty acids myristoleic acid 14∶1(11) were the most significantly overexpressed (45-fold) after 1hour of exposure to 4°C. As reported in the literature, these fatty acids play important roles in cold adaptability by supplying cell membrane fluidity, and by providing energy to cells. Analysis of water-soluble compounds revealed that isobutyrate, sarcosine, threonine and valine were more accumulated during exposure to 4°C. These metabolites might play a role in conferring cold acclimation to strain N_33_ at 4°C, probably by acting as cryoprotectants. Isobutyrate was highly upregulated (19.4-fold) during growth at 4°C, thus suggesting that this compound is a precursor for the cold-regulated fatty acids modification to low temperature adaptation.

## Introduction

Bacteria have developed many strategies at the transcriptional and post-transcriptional levels to enhance their abilities to withstand cold temperature stress. Some molecular responses to stress are general while others can be specific [1]. For instance, by using genome, cell physiology and transcriptome analyses, it has been shown that at −15°C, the permafrost bacterium *Planococcus halocryophilus* strain Or1, specifically regulates genes encoding for: the conversion of saturated fatty acids to unsaturated and branched fatty acids; the remodeling of cytoplasmic membrane and cell envelope features; transport systems, chaperone proteins, accumulation of cryoprotectant compounds, and transcriptional regulation [2]. The specific cold adaptive features in bacteria include global resource efficiency, amino acid substitution in cold-active enzymes and increased substrate transport systems [2], [3]. Low temperatures also regulate transcripts encoding for specific enzymes (e.g. catalase, glutathione S-transferase, Mo-molybdopterin cofactor biosynthesis and acetyl-CoA dehydrogenase) potentially involved in oxidative stress response by neutralizing toxic compounds such as reactive oxygen species (ROS) [2], [4]. A genome sequence analysis study of the psychrophilic Antarctic bacterium *Pseudoalteromonas haloplanktis* TAC125, suggested that elimination of the entire metabolic pathways involved in ROS generation, can protect the cell against the accumulation of deleterious dioxygen scavenging [5]. Like in many other cold-loving bacteria, the synthesis of lipid desaturases has also been detected in strain TAC125. These enzymes increase membrane fluidity, and protect the cell against dioxygen and detoxify the cells at low temperature. On the other hand, as a general response, housekeeping genes are also regulated by a variety of stressors including low temperature which allows these bacteria to down-regulate their metabolism to optimize general cell functions in order to withstand cold stresses. Other general response mechanisms include higher turnover of macromolecules, tighter maintenance of intercellular pH, greater osmotic regulation, motility, stopping biomass production and decreasing the activation energy before a pre-exponential growth phase [6]. Reduction of growth and suppression of the genes involved in translation and ribosomal biogenesis in *E. coli* have been observed and can be considered as a general response and an important strategy under stress conditions [7]. Transcriptional analysis of the Arctic *Mesorhizobium* N_33_ revealed the down-regulation of many housekeeping genes that encode for the cell envelope and outer membrane biogenesis functions, as well as cell motility and secretion at low temperature (unpublished data).

Studies on cold adaptations of the mesophilic [8]–[10] and psychrophillic bacteria [11], [12] indicated that they are widely heterogeneous in their genomes content and encompass broad ranges of complex network strategies to survive at low temperature. Most cold adaptation comprehensive studies were mainly performed by applying high-throughput genome sequencing [4], [5], [11] and other omics technologies such as proteomics [13], [14] and transcriptomics [15]. In *E. coli*, it has been shown that under different perturbations (cold, heat, lactose diauxie, and oxidative stress) the metabolite profiles are much more stress-specific when compared with transcriptomic changes [16].

In our studies, the transcriptional analysis of Arctic *Mesorhizobium* N_33_ at different cold temperature treatments has been informative (unpublished data). For instance, we observed that some amino acids, polyunsaturated fatty acids, and cryoprotectants changed significantly. Since transcriptomics studies only allow the evaluation of molecular adaptation mechanisms at the level of gene expression and did not reflect much higher specificity during early stress adaptation [16], we hypothesised that using metabolomics measurements will allow a better understanding of N_33_ cells response to its environmental changes [16]–[18]. Furthermore, metabolites are the end products of cellular processes, and they can be considered as a link between genotype (gene function) and phenotype [19]. It was previously shown that metabolic profiles could be good and specific indicators for monitoring cellular responses to biotic and abiotic stresses [18]. Nevertheless, our understanding of the metabolite content of cold adapted bacteria is very limited.

The psychrotrophic Arctic *Mesorhizobium* sp. N_33_ is the best known cold-adapted symbiotic N_2_-fixing rhizobium isolated from nodules of *Oxytropis arctobia*, an indigenous legume from Canada’s eastern Arctic [20]. Strain N_33_ has an intrinsic resistance to streptomycin and it has been conformable to genetic manipulations [21]–[23]. Strain N_33_, like its closely related strain N_31_, has a growth temperature range between 0°C and 30°C, and can establish an efficient symbiosis with the temperate forage crop sainfoin (*Onobrychis viciifolia*), forming nodules with nitrogenase enzyme (catalyzing the reduction of N_2_ to NH_3_^+^) active at 10°C [24]. This feature allows legumes to grow in soil that is cold and poor in nitrogen. Since as described earlier, bacteria respond to cold temperature stresses by modifying the level of expression of many genes influencing important cell functions, we hypothesized that in the arctic strain N33, the metabolites found in cell cultures or exposed to suboptimal temperatures, will also vary accordingly. Understanding the variation in N_33_ metabolites, will be helpful for future trials aiming at the elucidation of the previously observed cold adaptation of the nitrogenase activity in this Arctic bacterium [24].

Metabolites are very diverse in terms of their chemical structures and abundance [25]. Metabolomic analyses can be performed by using a variety of analytical tools [25]–[28]. In this study we applied both nuclear magnetic resonance (NMR) spectroscopy [29] and gas chromatography-mass spectrometry (GC-MS) techniques [25], [30] to determine and quantify the water soluble and lipid soluble metabolites [31] of the Arctic *Mesorhizobium* N_33_ subjected to suboptimal temperatures. These data were compared to cells grown at 21°C. Multivariate and univariate statistical analyses, and hierarchical clustering including heatmaps were applied to visualize, identify and compare compounds that were most strongly associated with low temperature adaptation. Our results showed that quantitative metabolomics profiling offers new insights into the chemical environment and metabolic adaptations used by the Arctic *Mesorhizobium* N_33_ at low temperature.

## Results and Discussion

### Experimental Design and Metabolic Profiles

**Figure 1:**
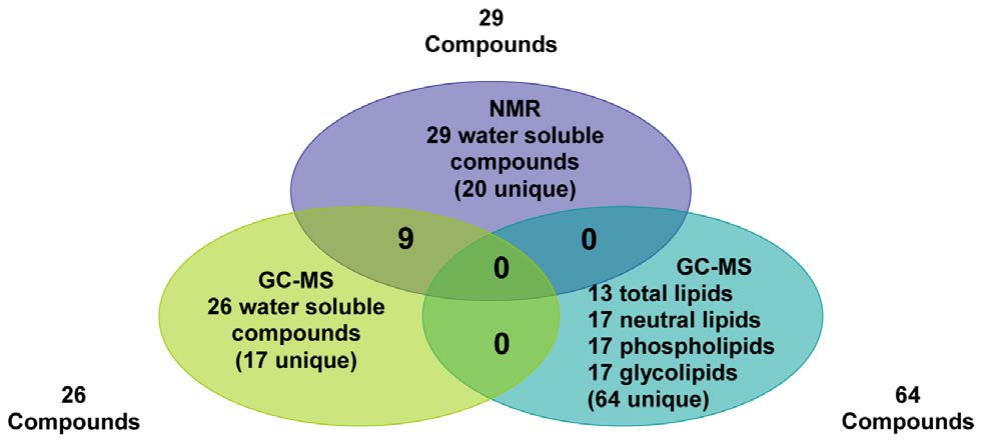
Venn diagram showing the overlap of *Mesorhizobium* N_33_ water soluble metabolites detected by NMR and GC-MS, and fatty acids from different lipids detected by GC-MS.

GC-MS and NMR were used for the identification and quantification of water soluble metabolites and fatty acids in cells of *Mesorhizobium* N_33_ growing at or exposed to, suboptimal temperatures as compared to cells grown at 21°C considered as control in this study. Water-soluble metabolites and lipids were extracted by phase separation using a biphasic system as described in the materials and methods section. NMR analyses of water soluble metabolites allowed the identification and quantification of 29 compounds (**Table 1**, and **Table S1**). With GC-MS, 26 water-soluble metabolites were identified, of which nine were also detected by NMR (**Table 1** and **Figure 1**).

**Table 1:** Water soluble metabolites of Arctic *Mesorhizobium* N_33_ detected by NMR and by GC-MS.

Metabolites identified byNMR	HMDB ID	Metabolites identified by GC-MS	HMDB ID
3-Hydroxybutyrate	HMDB00357	2,3-Dimethoxy phenylpyruvic acid	HMDB00205
Acetate	HMDB00042	4-aminobutyric acid	HMDB00112
Acetone	HMDB01659	Beta-Amino isobutyric acid	HMDB02166
**Alanine**	HMDB00161	Beta-Hydroxybutyric acid	HMDB01873
Choline	HMDB00097	Adenosine	HMDB00050
Ethanol	HMDB00108	**Alanine**	HMDB00161
Formate	HMDB00142	Arabino-Hexos-2-ulose	CHEBI:27973
**Glucose**	HMDB00122	Arabinofuranose	HMDB12325
Glutamate	HMDB03339	Cadaverine	HMDB02322
**Glycerol**	HMDB00131	Galactose	HMDB00143
Glycine	HMDB00123	Gluconic acid	HMDB00625
Isobutyrate	HMDB01873	**Glucose**	HMDB00122
Isoleucine	HMDB00172	**Glycerol**	HMDB00131
Lactate	HMDB00190	**Lysine**	HMDB00182
Leucine	HMDB00687	**Malonic acid**	HMDB00691
**Lysine**	HMDB00182	**Mannitol**	HMDB00765
**Malonic acid**	HMDB00691	Mannose	HMDB00169
**Mannitol**	HMDB00765	**Methionine**	HMDB00696
**Methionine**	HMDB00696	Methylaminobutyric acid	HMDB00039
N-Acetylglycine	HMDB00532	Methylmalonate	HMDB00202
N-Carbamoyl-β-alanine	HMDB00026	Oxalic acid	HMDB02329
Oxypurinol	HMDB00786	Phenylalanine	HMDB00159
Phenylacetate	HMDB00209	Putrescine	HMDB01414
Sarcosine	HMDB00271	Succinic acid	HMDB00254
Succinate	HMDB00254	**Tyrosine**	HMDB00158
Threonate	HMDB00943	**Valine**	HMDB00883
Threonine	HMDB00167		
**Tyrosine**	HMDB00158		
**Valine**	HMDB00883		

Compounds indicated in bold were detected by NMR and GC-MS.

HMDB: Human metabolome database (http://www.hmdb.ca/).

Mean concentrations (µM) of water-soluble metabolites (measured by NMR) and their standard deviation (SD) are shown in the supporting information (**Table S1**). Fatty acids from total, neutral, glyco- and phospho- lipids were identified and quantified using GC-MS. We were able to identify and quantify 13 types of fatty acids from total lipids, 17 from neutral lipids, 17 from phospholipids, and 17 from glycolipids. The complete list of fatty acids, mean concentrations (expressed as mole % of total fatty acids) and their standard deviation (SD) are shown in supporting information (**Tables S2, S3, S4**, and **S5**). A list of the 20 fatty acids identified in N_33_ is presented in **Table 2**.

**Table 2:** Fatty acids identified by GC-MS in cold exposed Arctic *Mesorhizobium* N33.

Fatty Acids	Common Name	IUPAC Name (Systematic)
**C12**	Lauric (Dodecanoic) acid	Dodecanoic acid
**C14**	Myristic (Tetradecanoic) acid	Tetradecanoic acid
**C14∶1(11)**	Myristoleic acid	Tetradecenoic acid
**C15**	Pentadecanoic acid	Pentadecanoic acid
**C16**	Palmitic acid	Hexadecanoic acid
**C16∶1 (7)**	Palmitoleic acid	Hexadecenoic acid
**C16∶1(9)**	Palmitoleic acid	Hexadecenoic acid
**C18**	Stearic acid	Octadecanoic acid
**C18∶1**	Oleic acid	Octadecenoic acid
**C18∶1(10)**	Oleic acid	Octadecenoic acid
**C18∶1(9)**	Oleic acid	Octadecenoic acid
**C18∶2(6,9)**	Linoleic acid	Octadecadienoic acid
**C18∶2(9,12)**	Linoleic acid	Octadecadienoic acid
**C19**	Nonadecanoic acid	Nonadecanoic acid
**C19∶1(10)**	Nonadeca-10(Z)-enoic acid	Nonadecenoic
**C20**	Arachidic acid	Eicosanoic acid
**C20∶1**	11-Eicosenoic acid	Eicosenoic acid
**C20∶1(11)**	11-Eicosenoic acid	Docosenoic acid
**C22∶1**	Erucic acid	Eicosenoic acid
**C22∶1(13)**	Cetoleic acid	Docosenoic acid

### Multivariate Statistical Analyses of Water Soluble Metabolites

**Figure 2:**
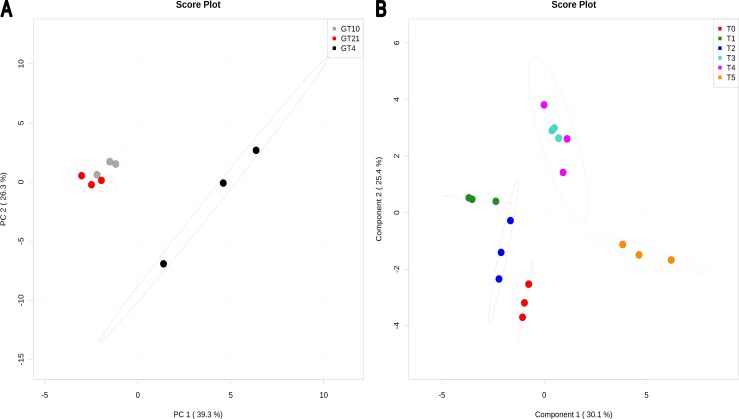
PCA and PLS-DA of water soluble metabolites found in *Mesorhizobium* N_33_ growing at constant temperatures or exposed to suboptimal 4°C. Growth temperatures: GT21 = 21°C (control); GT4 = 4°C; GT10 = 10°C. For all data, row-wise normalization was used by a pooled averaged reference samples (GT21 or T0). Data were auto scaled and log transformed. A: PCA analysis was performed based on 29 water soluble metabolites. B: PLS-DA plot showing grouping of compounds (permutation test, P<0.01) according to the different time of exposure to a suboptimal 4°C temperature. T0 = 21°C (reference), T1 = 2 min; T2 = 4 min; T3 = 8 min; T4 = 60 min; T5 = 240 min exposure to 4°C of cells grown at 21°C. The optimal PLS-DA model for water soluble metabolites data (measured by NMR) used the top four components with a Q^2^ = 0.78.

Principal components analysis-PCA (**Figure 2A**) and heatmap visualization (**Figure S2**) show that growth temperature significantly affects water soluble metabolites in *Mesorhizobium* N33. Cells grown at 10°C had water soluble metabolites contents, comparable to that of the control cells grown at 21°C. Nevertheless, slight differences in the level of metabolites accumulation were observed between the two temperatures. Cells grown at 4°C clustered in a distinct group indicating that at this suboptimal temperature, changes in metabolites levels (up and down) were more pronounced. Out of 29 water soluble metabolites, 19 showed a high accumulation in at least two independent biological repetitions and 10 compounds were suppressed during acclimation to 4°C. Isobutyrate, sarcosine, threonine, and valine were up-regulated in all three biological replicates at 4°C (**Figure S2**), suggesting that these compounds might be required for energy conservation [2], [16]. The significant compounds of N_33_ water soluble metabolites after different times of exposure to suboptimal 4°C temperature (T1 to T5; 2 to 240 min), are shown based on PLS-DA (partial least squares discriminate analysis) (**Figure 2B**) and heatmap visualization (**Figure S3**). The significant (P<0.01) permutation test confirmed the PLS-DA analysis. The optimal PLS-DA model for water soluble metabolites data (measured by NMR) used the top four components with a Q^2^ = 0.78. Results revealed that some metabolites accumulated while others decreased after different times of exposure to 4°C compared to the control (T0 = 21°C). The most important compounds are indicated in **Figure S2** and they include: sarcosine, glycine, lactate, glutamate, glucose, methionine, 3-hydroxybutyrate, acetate, tyrosine, and isoleucine. The increase in amino-acid levels might result, at least in part, from an increase in protein degradation [32] necessary for the elimination of abnormal proteins resulting from stress conditions. Abundance of the amino-acids might also be due to the demand for the synthesis of new important proteins (chaperones) under stress conditions [16], [33]. A genomic study has supported the thermal flexibility apparent from amino acid distribution in the permafrost bacterium (*Planococcus halocryophilus* strain Or1) [2].

### Multivariate Statistical Analyses of Fatty Acids from Neutral Lipids

**Figure 3:**
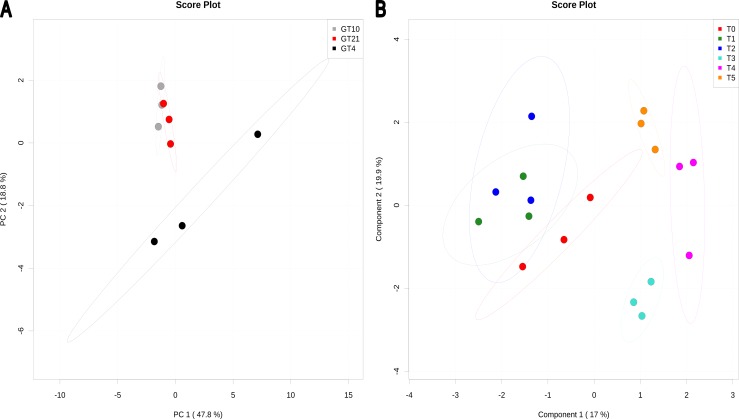
PCA and PLS-DA of fatty acids from neutral lipids present in *Mesorhizobium* N_33_ growing at constant temperatures or exposed to suboptimal 4°C. Growth temperatures: GT21 = 21°C (control); GT4 = 4°C; GT10 = 10°C. For all data, row-wise normalization was realized with a pooled averaged reference sample (GT21 or T0), and data were auto scaled and log transformed. A: PCA analysis was performed based on 17 fatty acids. B: PLS-DA plot showing compound changes (permutation test significant at P<0.01) after exposure for different time to a suboptimal 4°C temperature. T0 = 21°C (reference), T1 = 2 min; T2 = 4 min; T3 = 8 min; T4 = 60 min; T5 = 240 min exposure to 4°C of cells grown at 21°C. The optimal PLS-DA model for fatty acids from neutral lipids used the top two component with a Q^2^ = 0.37.

PCA analysis (**Figure 3A**) and heatmap visualization (**Figure S4**) indicated that neutral lipids in cells of Arctic strain N_33_ grown at 10°C exhibited fatty acids content comparable to the one observed when cells were grown at 21°C. However a distinct content was obtained when N_33_ was cultivated at the suboptimal 4°C temperature. Out of 17 measured fatty acids, linoleic acid 18∶2(6,9) showed higher accumulation in cells grown at 4°C. Many metabolites have shown same change trends at 10 and 21°C (**Figure S4**). The significant fatty acids compounds from neutral lipids after different times of exposure to suboptimal 4°C temperature, are shown according to PLS-DA analysis (**Figure 3B**), and heatmap visualization (**Figure S5**). The permutation test was statistically significant (P<0.07) which confirmed the results of PLS-DA analysis. The optimal PLS-DA model for fatty acid of neutral lipids used the top two components with a Q^2^ = 0.37.

### Multivariate Statistical Analysis of Phospholipids Fatty Acids Content

**Figure 4:**
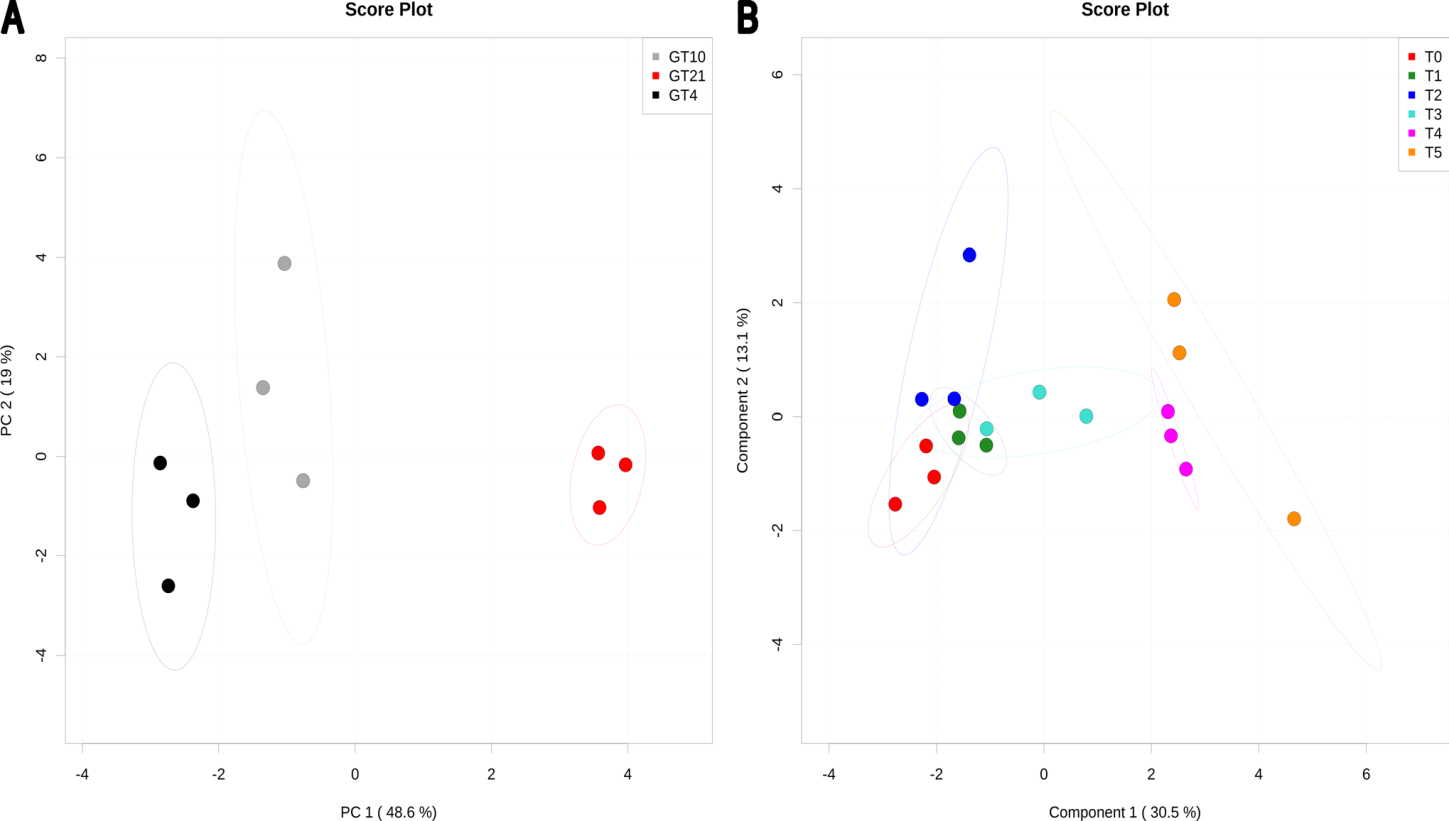
PCA and PLS-DA of fatty acids from phospholipids present in *Mesorhizobium* N_33_ growing at constant temperatures or exposed to suboptimal 4°C. Growth temperatures: GT21 = 21°C (control); GT4 = 4°C; GT10 = 10°C. For all data, row-wise normalization was used by a pooled averaged reference samples (GT21 or T0), and data were auto scaled and log transformed. A: PCA analysis was performed on 17 fatty acids. B: PLS-DA plot showing the compound changes (permutation test, P<0.01) at different times of exposure to suboptimal 4°C. T0 = 21°C (reference), T1 = 2 min; T2 = 4 min; T3 = 8 min; T4 = 60 min; T5 = 240 min to 4°C of cells grown at 21°C. The optimal PLS-DA model for fatty acids from phospholipids used the top five component with a Q^2^ = 0.74.

The PCA analysis (**Figure 4A**) of the phospholipids fatty acids content of N_33_ growing at constant temperatures and heatmap visualization (**Figure S6**) show distinct trend of metabolite changes at each growth temperature tested (21°C, 10°C and 4°C). Out of 17 measured fatty acids from phospholipids, the linoleic acids 18∶2(9,12), 18∶2(6,9), palmitoleic acid 16∶1(7), and cetoleic acid 22∶1(13) showed higher accumulation in N_33_ growing at 4°C (**Figure S6**). Significant changes in phospholipids fatty acids after different times of exposure to suboptimal 4°C temperature revealed by PLS-DA analysis (**Figure 4B**), and heatmap visualization are shown in **Figure S7**. The significant permutation test (P<0.01) confirms the results of PLS-DA analysis. The optimal PLS-DA model for phospholipids fatty acids used the top five component with a Q^2^ = 0.74. The PLS-DA and heatmap show increases or decreases in metabolite after each time of exposure (from T0 to T5) to 4°C.

### Multivariate Statistical Analysis of Fatty Acids from Glycolipids

**Figure 5:**
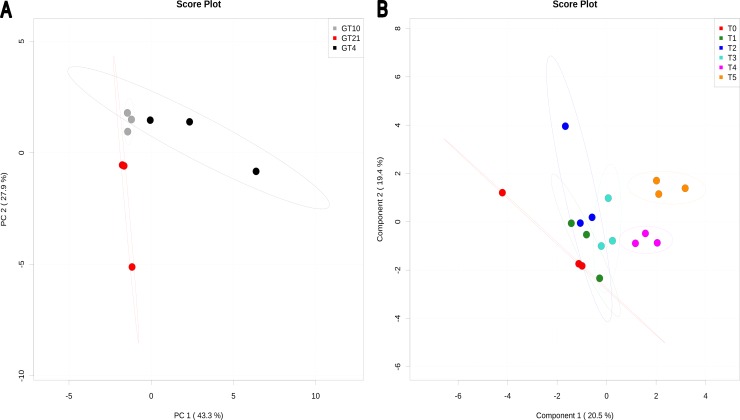
PCA and PLS-DA of fatty acids from glycolipids present in *Mesorhizobium* N_33_ growing at constant temperatures or exposed to suboptimal 4°C. Growth temperatures: GT21 = 21°C (control); GT4 = 4°C; GT10 = 10°C. For all data, row-wise normalization was used by a pooled averaged reference samples (GT21or T0), and data were auto scaled and log transformed. A: PCA analysis was performed on 17 fatty acids. B: PLS-DA plot showing compound changes (permutation test, P<0.01) at different times of exposure to suboptimal 4°C. T0 = 21°C (reference), T1 = 2 min; T2 = 4 min; T3 = 8 min; T4 = 60 min; T5 = 240 min exposure to 4°C of cells grown at 21°C. The optimal PLS-DA model for fatty acids from glycolipids used the top three component with a Q^2^ = 0.75.

The PCA analysis (**Figure 5A**) of the glycolipids fatty acids present in N_33_ growing at constant temperatures and heatmap visualization (**Figure S8**) show different trends of metabolite changes. N_33_ cells growing at 4°C show a distinct trend as compared to cells cultivated at 21°C, whereas an intermediate level of metabolite changes is observed with cells growing at 10°C. Out of 17 measured fatty acids from glycolipids, linoleic acid 18∶2(6,9), nonadecanoic acid (C19), myristic (tetradecanoics) acid (C14) concentration increased at 4°C, and linoleic acid 18∶2(6,9) increased at 10°C. These fatty acids were present at lower concentrations in cells cultivated at 21°C (**Figure S8**). The significant fatty acids compounds of glycolipids after different times of exposure to suboptimal 4°C temperature identified by PLS-DA analysis (**Figure 5B**) and heatmap visualization (**Figure S9**). The significant permutation test (P<0.01) confirms the results of PLS-DA analysis. The optimal PLS-DA model for fatty acid from glycolipids used the top three component with a Q^2^ = 0.75. The PLS-DA and heatmap showed that time of exposure to suboptimal 4°C significantly influenced the concentration of glycolipids fatty acids in the arctic strain N_33_ (**Figure 5B**).

### Multivariate Statistical Analysis of Fatty Acids from Total Lipids

**Figure 6:**
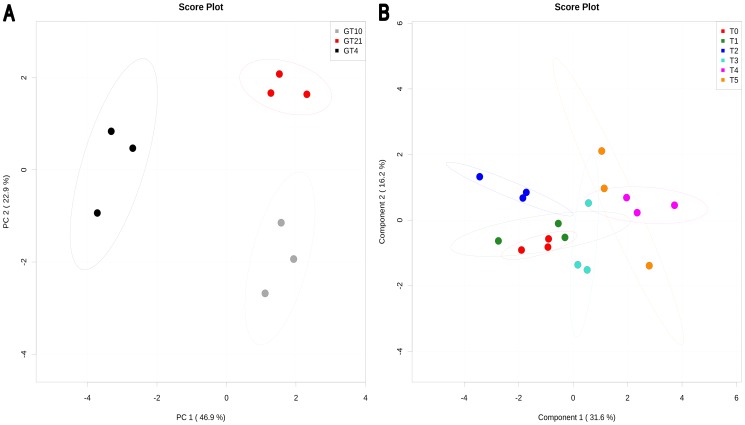
PCA and PLS-DA of fatty acids from total lipids present in *Mesorhizobium* N_33_ growing at constant temperatures or exposed to suboptimal 4°C. Growth temperatures: GT21 = 21°C (control); GT4 = 4°C; GT10 = 10°C. For both groups of data, row-wise normalization was used by a pooled averaged reference samples (GT21orT0), and data were auto scaled and log transformed. A: PCA analysis was performed on 13 fatty acids total. B: PLS-DA plot of total fatty acids data from GC-MS shows significant trends of the separation of compounds changes (permutation test, P<0.01) at different times of cold treatment conditions T0 = 21°C (reference), T1 = 2 min; T2 = 4 min; T3 = 8 min; T4 = 60 min; T5 = 240 min exposure to 4°C of cells grown at 21°C. The optimal PLS-DA model for fatty acids from total lipids used the top one component with a Q^2^ of 0.31.

The PCA analysis (**Figure 6A**) and heatmap visualization (**Figure S10**) show that the total fatty acids content of N_33_ is significantly affected by growth temperature. Distinct trends of metabolite changes at 21°C, 10°C and 4°C were observed by heatmap visualization (**Figure S10**). Out of 13 total fatty acids, at least 6 compounds showed accumulation at 4°C and 7 others decreased. These patterns were opposite in N_33_ growing at 21 and 10°C. Significant fatty acids from total lipids after different times of exposure to suboptimal 4°C temperature revealed by PLS-DA analysis (**Figure 6B**), and heatmap visualization is shown in **Figure S11**. The significant permutation test (P<0.01) confirms the results of the PLS-DA analysis. The optimal PLS-DA model for fatty acids from total lipids used only the top component with a Q^2^ of 0.31. The PLS-DA and heatmap showed different trends of metabolite changes for each time of exposure to 4°C.

Unsupervised PCA analysis of fatty acids from the 4 lipid classes, disclosed that the metabolite profiles of N_33_ cells grown at 21°C, 4°C or 10°C are each clustered into a distinct group, contrary to cells grown at 21°C and exposed to 4°C for 2 to 240 minutes, thereby suggesting a specific metabolic acclimation to cold.

To distinguish and better understand the detailed information arising from the multivariate analyses, and to identify metabolite features that are significantly different between each treatment and the control, univariate statistical analysis (*i.e*. t-tests, determining the fold change (FC) and volcano plots) were performed. These analyses were applied to five different metabolite data sets (total fatty acids, and fatty acids from neutral, glyco- and phospho- lipids, and water soluble compounds) and are presented below.

### Univariate Statistical Analyses of Fatty Acids

**Figure 7:**
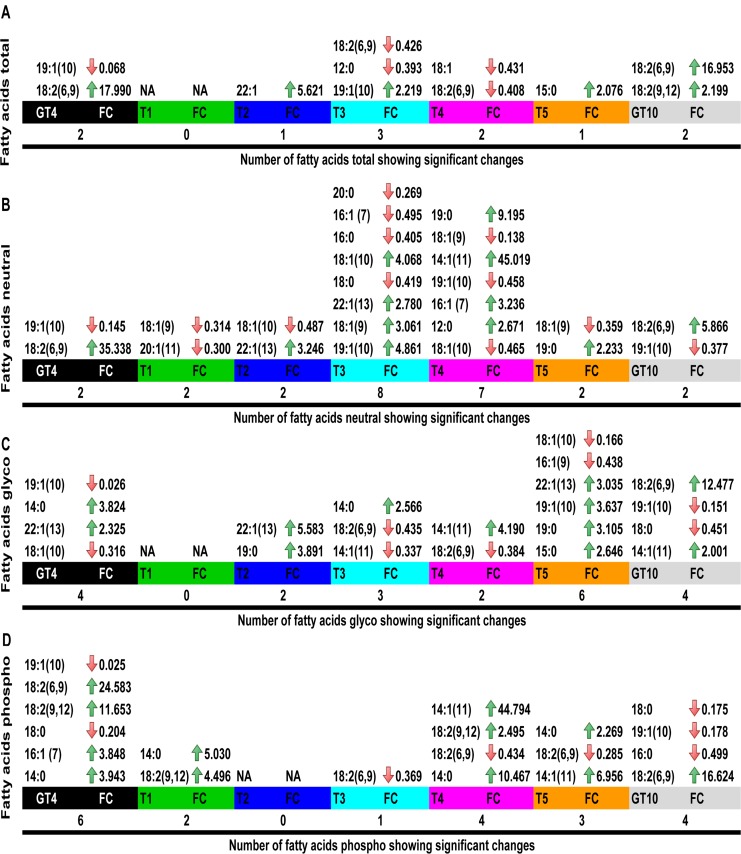
Significant changes (P≤0.05, FC ≥2) of fatty acids in *Mesorhizobium* N_33_ exposed to cold. GT4 =  Growth at 4°C; T1 = 2 min, T2 = 4 min, T3 = 8 min, T4 = 60 min, T5 = 240 min exposure to 4°C of cells grown at 21°C; GT10 =  Growth at 10°C. Observed significant changes: A: fatty acids from total lipids; B: fatty acids from neutral lipids; C: fatty acids from glycolipids and D: fatty acids from phospholipids. Arrows indicate change in concentration: green-increase, red-decrease. FC =  fold changes; NA =  no significant change.

When grown at 21°C the arctic M*esorhizobium* N_33_ had a total fatty acid content formed mainly by the following lipids (mole % of total lipids): 18∶1 (54%); 16∶0 (26%); 18∶0 (13.5%) and 19∶1(10) (3.19%) (**Table S2**). Monounsaturated 18∶1, along with the saturated 16∶0 and 18∶0 fatty acids are the major lipids found in rhizobia grown at optimal temperature [34]–[37]. The fatty acids identified by GC-MS which showed significant (P≤0.05; FC ≥2) concentration shifts at low temperatures are shown in **Figure 7**.

The maximum increases in fold-change (FC) were observed when N_33_ was grown at 4°C or 10°C (GT4 or GT10). In fact, when grown at 4°C and 10°C, N_33_ contained respectively 18 and 17 times more linoleic acid 18∶2 (6, 9) than cells grown at 21°C. The concentration of the fatty acid 19∶1(10) significantly decreased in cells grown at 4°C. N_33_ cells growing at 21°C (T0) then exposed to 4°C for 2 min (T1) did not exhibit any significant change in fatty acids measured from total lipids. However, when exposed to cold for 4 or 8 min (T2 and T3), 5.6 and 2.2 fold increases were observed for fatty acids 22∶1 and 19∶1(10) (**Figure 7A**).

Univariate statistical analysis indicated that the concentration of 13 (5 saturated and 8 unsaturated) fatty acids from neutral lipids significantly (P≤0.05, FC ≥2) changed in N_33_ cells submitted to cold treatments (**Figure 7B**). Overall, 12 accumulations and 13 reductions in fatty acids from neutral lipids were observed with all cold treatments. As observed with fatty acids of total lipids, at 4°C N_33_ cells contained more (FC = 35) fatty acid 18∶2 (6, 9) than cells of the control (growth at 21°C). Comparable results were observed at 10°C (FC = 6). An important increase (FC = 45) in the concentration of fatty acid 14∶1 (11) was observed only in N_33_ cells exposed to 4°C for1 h. Under the same condition, the highest fold change ratio (FC = 9) was observed for the saturated fatty acid 19∶0. The most important decrease in concentration was observed for the saturated fatty acid 20∶0 (FC = 0.27), in N_33_ cells after 8 min exposure to 4°C (**Figure 7B**).

The significant changes in fatty acids from glycolipids of *Mesorhizobium* N_33_ exposed to cold temperature are shown in **Figure 7C**. Out of the 17 fatty acids detected from glycolipids (**Figure 1** **and Table S4**), 4 saturated and 6 unsaturated changed concentrations after cold treatments. Significant (P≤0.05, FC ≥2) levels of accumulation resulting from exposure to cold, ranged from 2.32 FC for fatty acid chain 22∶1 (13) at 4°C to 12.5 FC for 18∶2 (6, 9) in N_33_ growing at 10°C.

Reduction in the concentration of fatty acids from glycolipids in N_33_ ranged from a FC of −38.46 for fatty acid 19∶1 (10) at 4°C to a FC of −2.22 for fatty acid 18∶0 for growth at 10°C. As revealed by univariate statistical analysis 8 (5 unsaturated and 3 saturated) out of 17 fatty acids from phospholipids identified in *Mesorhizobium* strain N_33_ (**Figure 1** **and Table S5**), showed significant (P≤0.05, FC ≥2) changes in concentrations after cold perturbations. N_33_ cells grown at 4°C or at 10°C, contained respectively 24.5 and 16.6 times more linoleic acid 18∶2(6, 9) than cells growing at 21°C (**Figure 7D**). Phospholipids from strain N_33_ cultivated at 4°C, contained substantially more fatty acids 16∶1(7) (FC = 3.8), 14∶0 (FC = 3.9), and 18∶2(9, 12) (FC = 11.6) than the control. In N_33_ cells exposed to 60 min 4°C an important increase in fatty acids 14∶0 (FC = 10.4) and 14∶1 (11) (FC = 44.7) were observed (**Figure 7D**). The concentration of fatty acid 19∶1(10) in N_33_ significantly decreased in cells cultivated at 4°C (FC = −40) and at 10°C (FC = −5.55; **Figure 7D**).

This study shows that some saturated and many unsaturated fatty acids of the Arctic *Mesorhizobium* N_33_ were affected by suboptimal temperatures. Saturated fatty acids have the least steric interference with neighboring methylene groups, and so they may be used to enhance the rigid structure of the membrane and protect the cell conformation. Protection of psychrophilic bacteria from cold temperatures by the overproduction of polyunsaturated fatty acid was previously demonstrated by using transcriptional and biochemical analyses [38], [39]. Unsaturated fatty acids have kinks which limit acyl-chain packing and cause a melting point reduction (melting point for linoleic acid 18∶2(9, 12) is −8.5°C). This feature improves cell membrane fluidity at low temperatures and the increased production of unsaturated fatty acids appears to be one mechanism for the low temperature adaptation in Arctic *Mesorhizobium* N_33_. Our results corroborate previous observations indicating that rhizobia increase the production of unsaturated fatty acids during growth at cold temperatures [35], [37].

Interestingly, a marked effect of temperature on the fatty acid composition in N_33_ was reflected by the different trends observed, when cells were grown at 4°C or 10°C (**Figures 3A** **to** **6A**). Nevertheless, at both temperatures, N_33_ tends to produce significantly more linoleic acid 18∶2 (6, 9) and less of 19∶1(10) than the control cells for the different types of fatty acids studied. Changes in fatty acid composition following different time of exposure to cold temperatures were more diverse.

### Univariate Statistical Analysis of Water Soluble Metabolites

**Figure 8:**
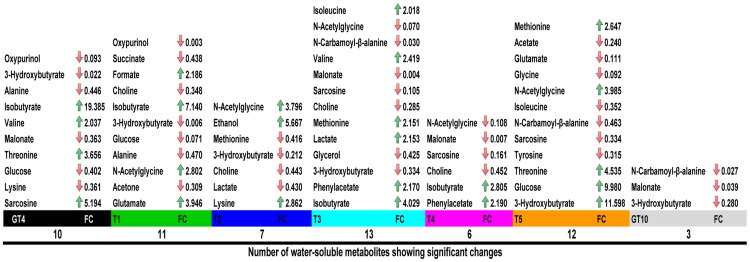
Significant changes (P≤0.05, FC ≥2) of water soluble metabolites in N_33_ exposed to cold. GT4 =  Growth at 4°C; T1 = 2 min, T2 = 4 min, T3 = 8 min, T4 = 60 min, T5 = 240 min exposure to 4°C of cells grown at 21°C, GT10 =  Growth at 10°C. Arrows indicate change in concentration: green-increase, red-decrease. FC =  fold changes; NA =  no significant change.

The significant changes (P≤0.05, ratio≥2) in the water-soluble metabolites content of *Mesorhizobium* N_33_ subjected to different suboptimal temperature treatments were identified by NMR and are summarized in **Figure 8**. Univariate statistical analysis revealed that the concentration of several compounds were significantly (P≤0.05, ratio≥2) altered by cold. In comparison to cells grown at 21°C, N_33_ grown at 4°C contained significantly more valine, threonine, sarcosine and isobutyrate with FC ranging from 2 to 19.4 (**Figure 8** **and Table S1**). Seven water soluble compounds showed a significant concentration decrease at 4°C, but the most important were observed with 3-hydroxybutyrate and oxypurinol. Fewer changes were observed in N_33_ grown at 10°C compared to 21°C. In fact only 3 compounds changed concentrations and N-carbamoyl-β-alanine displaying the lowest FC (**Figure 8**). When grown at 21°C and then exposed to 4°C for different periods of time, in general, N_33_ showed more decreases than increases in water soluble metabolites, when compared to the control cells grown at 21°C (**Figure 8** **, Table S1**).

The metabolite with the highest fold change for all temperature conditions was isobutyrate, with a 19.4 fold change increase found in N_33_ growing at 4°C (GT4). The greatest reduction in water-soluble metabolites was observed at some conditions with a 0.003 fold change. This lowest level of metabolites fold change might be either the result of being below the instrument detection limit or that the level of the metabolite production has been very low compared to control conditions (N_33_ grown at 21°C). As a general rule, NMR methods are not particularly sensitive, but using this technique reduces the loss of compounds that may occur during sample preparation [29]. Combining GC-MS and NMR can compensate for the lack of coverage of each platform, but both are still insufficiently sensitive to cover all metabolites. It has been suggested that LC-MS or DI-MS (direct injection of metabolites spectrometry) might be the best methods for metabolomics because of their high sensitivity despite their bias against hydrophilic metabolites [26].

Overall, our metabolomic study of the Arctic *Mesorhizobium* N_33_ using GC-MS and NMR was able to identify 110 compounds involved in central carbon metabolism, essential biosynthetic pathways, secondary metabolism and lipids under different low temperature treatments. GC-MS could measure 64 fatty acids (**Figure 1** **, Tables S2, S3, S4 and S5**) and identify a variety of amino acids and organic acids (**Table 1**). NMR spectroscopy provided complementary information by enabling the quantification of 29 water-soluble metabolites (**Figure 1** **,** **Table 1**).

The metabolomic analysis of Arctic strain N_33_ indicated that among the lipid-soluble compounds, poly-unsaturated linoleic acids 18∶2(9, 12) and 18∶2 (6, 9) were the most abundant fatty acids present in cells grown at 4°C or 10°C, as compared to the control cells growing at 21°C. The mono-unsaturated fatty acid (myrestic acid) 14∶1(11) from phospho- and neutral lipids was the most significantly overexpressed (45-fold change) after exposure for 60 min to 4°C. These fatty acids are known to provide physical membrane flexibility adaptation and to supply energy to cells [40].

Analysis of water-soluble compounds revealed that isobutyrate, sarcosine, therionine and valine increased during growth at 4°C and after exposure for different times to 4°C, of cells initially grown at 21°C (T0). Among the water-soluble metabolites, isobutyrate was highly upregulated (19.4-fold) in cells grown at 4°C, suggesting that this compound is a precursor for the cold-regulated fatty acid modification to low temperature adaptation [41]. We have observed that some metabolites decreased in N_33_ under cold conditions. This might be caused by growth cessation or reduction, which is an important strategy to adjust cellular physiology to cold stresses [16].

Sarcosine (N-methylglycine) is an intermediate and a by-product of glycine synthesis and degradation. Sarcosine oxidase demethylates sarcosine to glycine [42]. It can be derived from the catabolism of betaine, and metabolism of choline, betaine and sarcosine may be linked together [4]. In addition, sarcosine has a significant role in the glutathione metabolic pathway and is a cryoprotectant in psychrophilic bacteria grown at low temperature [5]. These metabolites might have a substantial impact in conferring cold resistance ability to the strain N_33_ at 4°C by potentially acting as cryoprotectants. Accumulation of cryoprotectants and the production of unsaturated and short chain fatty acids are considered examples of specific metabolic responses to low temperatures in psychrophillic bacteria as previously shown by transcriptomic and genomic sequencing [4]. Sarcosine is also involved in low temperature osmoadaptation [43] and can be used as a source of carbon, nitrogen and energy [4]. Genome sequencing analysis of the psychrophilic bacterium *Colwellia psychrerythraea* 34H using genomic and proteomic methodologies [4], and phylogenetic diversity and metabolic potential in a glacier metagenome study revealed that many genes are involved in the synthesis of cryo-osmoprotectants such as glycine, betaine, choline, sarcosine, and glutamate [44]. The cryo-protectants (metabolites and proteins) suppress the aggregation of cellular proteins, stabilize phospholipid bilayers, prevent or reduce ice-crystal formation and freezing damage in bacterial cells at low temperature [45]–[47]. The significantly regulated water and lipid soluble compounds are mainly involved in energy conservation, carbon, protein, nucleic acid, fatty acid, and cryoprotectant biosynthesis. Energy conservation is an essential part of energy stress responses and is associated with many types of stress reactions [5], [16].

The metabolite profiles of the Arctic *Mesorhizobium* N_33_ and the changes seen in both metabolite abundance and composition at different cold conditions show that the biochemical changes allowing bacterial cells to tolerate cold is complex. It also suggests that several mechanisms are involved in cold acclimation in the Arctic strain N_33_.

In conclusion, our metabolomic study, using GC-MS and NMR, showed that the Arctic *Mesorhizobium* N_33_ regulates the levels of many compounds, and displays many molecular changes related to cold tolerance.

To identify the pathways and confirm the pattern of important compounds of Arctic bacterium N_33_ cold adaptation ability, specific isotope dilution GC-MS or fluxomics techniques are required for accurately quantifying the metabolites in strain N_33_ under cold condition. Further investigations combining different omics technologies such as proteomics, genomics, transcriptomics, metabolomics, are required to provide a more complete system biology perspective [18] for a better understanding of the complex mechanisms of cold adaptation in the Arctic *Mesorhizobium* N_33_.

## Materials and Methods

### Strain Cultivation, Experimental Design and Sample Collections

Frozen glycerol stocks of Arctic *Mesorhizobium* sp. strain N_33_ [20] were used to inoculate 20 ml yeast mannitol broth (YMB) medium [48] containing 200 µg ml^−1^ streptomycin, incubated at 21°C for 5 days on a rotary shaker (180 rpm). The purity of the cells was monitored by plating on solid yeast mannitol agar (YMA) medium containing 200 µg ml^−1^ streptomycin and 25 µg ml^−1^ Congo red [49] after 4 days of incubation at 21°C. Subsequent purity test of the strain was performed with a nodulation test on *Onobrychis viciifolia* (sainfoin) [48] and by 16S-rDNA sequencing [50].

A total of 8 conditions with 3 biological replicates were used in this experiment. After 3 days of growth at 21°C in YMB, 100 µL of pure fresh N_33_ cells were used to inoculate 500 ml Erlenmeyer flasks containing 100 ml YMB. Flasks were incubated on a rotary shaker (180 rpm) until cells reached the mid-exponential log phase (OD_600_ = 0.4–0.6), under three growing temperature conditions (21°C = control, 4°C, and 10°C; GT21, GT4 and GT10). For the cold stress treatments, mid-log phase cells grown at 21°C (T0) were exposed to 4°C for different times (T1 = 2 min, T2 = 4 min, T3 = 8 min, T4 = 60 min, T5 = 240 min; **Figure S1**) in a rotary shaker water bath (180 rpm). The samples were immediately transferred into pre-chilled (4°C) Falcon tubes then centrifuged (10,000 *g*) at 4°C for 5 min. The pellets (100 mg) were washed once with cold TES buffer [51] to eliminate extra-cellular polysaccharides and centrifuged again. The cells were immediately quenched in liquid nitrogen [52] and stored at −85°C. Each 100 ml inoculated YMB provided approximately 100 mg fresh cells. In order to provide enough cell pellets, the experiments were replicated 10 times for each treatment. The pellets from 10 individual flasks per treatment were pooled together before the extraction process and considered as one biological replicate.

### Extraction of Lipids and Water-Soluble Metabolites

Bacterial pellets (0.12 g dry weight) were transferred into a 12 ml screw capped glass vials. Lipids were extracted from the bacterial pellets according to the method of Bligh and Dyer [53]. Pellets were homogenized with the Tissue-Tearor (Biospec Products, U.S.A) in 10 ml of 2∶1(v/v) chloroform: methanol and shaken thoroughly at 150 rpm for 2 h. The extractant was centrifuged at 3000 rpm for 30 min and the supernatant was transferred into a separate vial. The residue was further extracted with 10 ml of 2∶1 (v/v) chloroform: methanol, centrifuged again and both supernatants were pooled. Water soluble metabolites and the lipids were extracted by phase separation of a biphasic system, generated by the addition of one quarter volume of 0.88% potassium chloride solution to the chloroform: methanol extract. This mixture was shaken thoroughly for 30 min and centrifuged. The upper aqueous water-soluble metabolite layer obtained was carefully pipetted into a separate tube and purged with nitrogen gas to remove traces of the solvent. The lower organic lipid layer was evaporated under nitrogen. Lipids were then resuspended in hexane and immediately used or stored under nitrogen at −20°C. The upper aqueous fraction mainly comprised of water soluble metabolites was subsequently frozen in liquid nitrogen and lyophilized.

Total lipid extracts were further separated into neutral lipids, glycolipids and phospholipids classes on LC-Silica Sep Pak cartridges (Supelco) as described by Lynch and Steponkus [54]. Calculated amount of total lipid extract (10–15 mg) dissolved in 1 ml chloroform were transferred to the cartridge. Once the sample had entered the packing, residual sample was washed into the column using 2 ml chloroform followed by an additional 10 to 12 ml of the same solvent to elute neutral lipids. The glycolipids were eluted by the addition of 15 ml acetone: methanol (9∶1 v/v) while phospholipids were sequentially eluted using 10 ml methanol. The fractions were dried under nitrogen, resuspended in hexane and immediately used or stored under nitrogen at −20°C.

### Preparation of Fatty Acid Methyl Esters (FAMEs)

Fatty acid methyl esters (FAMES) were prepared according to Christie [55]. A known amount of lipids in 2∶1 chloroform: methanol (v/v) was mixed with an internal standard (heptadecanoic acid C17∶0, Sigma-Aldrich). The mixture was evaporated under nitrogen gas and 1 ml of the methylating reagent (2% sulphuric acid in methanol, v/v) was added. The mixture was incubated at 80°C for 1 h, cooled on ice for 10 min after incubation and neutralized by adding 0.5 ml of a 0.5% sodium chloride solution. FAMEs were extracted twice by vortexing after the addition of 2 ml aliquots of hexane. The two layers were allowed to separate and the upper hexane layer was recovered, and subjected to gas chromatographic analysis for quantification of fatty acids.

### Gas Chromatographic Analysis of FAMEs

Gas chromatographic analysis of FAMEs was done using heptadecanoic acid as the internal standard. The analysis was performed on an Agilent 6890N gas chromatography instrument coupled with an Agilent MS-5975 inert XL mass selective detector (Agilent technologies) using the electron impact (EI) ionization mode. Separation of fatty acids was achieved by injecting 2 µL of the FAMEs in a 5% phenyl 95% dimethylpolysiloxane column DB5 (Agilent J & W Scientific, 30×0.25 mm×0.25 µm). Splitless injection was performed with a constant carrier gas (helium) flow of 1 ml/min. Inlet temperature and transfer line temperatures were set at 250 and 280°C respectively. The temperature programming was as follows: an initial isotherm at 70°C was held for 1 min, raised to 76°C at a rate of 1°C/min, and from 76 to 310°C at a rate of 6.1°C/min. The MS ion source temperature was 230°C and the Quadrupole temperature was 150°C. Peak identification of fatty acids was carried out by comparison of chromatogram with mass spectral library (NIST) and against the retention times and mass spectra of the Supelco 37 component FAME mix (Sigma-Aldrich, St Louis, MO, USA).

### GC-MS Method for Identification of Water-Soluble Metabolites

To derivatize the metabolites for GC-MS analysis, 40 µL of methoxyamine hydrochloride (Sigma-Aldrich) in pyridine was added to the water-soluble extracts and incubated at room temperature for 16 hours. Then 50 µL (N-Methyl-N-trifluoroacetamide) MSTFA with 1% TMCS (Trimethylchlorosilane) derivatization agent were added and incubated at 37°C for 60 minutes on a hotplate. The samples were vortexed twice throughout the incubation period to ensure complete dissolution. Samples were refrigerated at 4°C for no longer than 48 hours before analysis in order to avoid any degradation of the derivatized compounds.

Derivatized extracts were analyzed using an Agilent 7890-5975C GC-MS instrument operating in an Electron Impact (EI) ionization mode. For GC-MS analysis, 2 µL of the derivatized samples were injected using a split/splitless injector with a split ratio of 5∶1 onto a HP-5MS capillary column (30 m×250 µm×0.25 µm). The helium carrier gas was set to a flow rate of 1 mL/min and the initial oven temperature was set to 70°C. The temperature was increased at 1°C/min to 76°C, and then at 6.1°C/min to 310°C. The total run time was 45 minutes. The full scan mode of the quadrupole MS was used at a mass range of 50–500 m/z, with a solvent delay of 6 minutes. The MS ion source temperature was 230°C and the Quadrupole temperature was 150°C. In GC-MS, a faster scan speed generally provides more data points across a chromatographic peak, but it tends to lower the ion statistics. In contrast, a slower scan rate produces few scans over the peak and results in better spectra. The scan speed of our quadrupole MS was optimized over a number of samples and it was found that a relatively slow scan rate of 1.7 scans gave the best results.

The AMDIS spectral deconvolution software (Version 2.62) from NIST (National Institute of Standards and Technology) was used to process the total ion chromatogram and the EI-MS spectra of each GC peak. After deconvolution, the purified mass spectrum of each of the trimethylsilated metabolites was identified using the NIST MS Search program (version 2.0d) which was linked to the NIST mass spectral library (2005). Retention Indices (RIs) were calculated using an external alkane standard. Metabolites were identified not only by matching the EI-MS spectra with the those of reference compounds from NIST library, but also by matching the experimental RI of each metabolite with an in-house RI library (containing 312 TMS-derivatized metabolites) developed in our laboratory [26].

### NMR Sample Preparation

To identify and quantify the water-soluble metabolites, the evaporated water soluble fraction from different samples (∼42.8 mg/ml extracted cells) was dissolved in 500 µL of 50 mM NaH_2_PO_4_ buffer pH 7. Thirty five µL of D_2_O and 15 µL of a buffer solution (0.5 mM DSS (disodium-2,2-dimethyl-2-silapentane-5-sulphonate) and 0.47% NaN_3_ in H_2_O) and 1 mM Imidazole were added to the sample. The sample amount in the final assay volume (350 µL) was 15 mg. The sample solution was vortexed for 1 minute, sonicated for 30 minutes, and transferred to a standard Shigemi microcell NMR tube for subsequent spectral analysis.

### NMR Spectroscopy

All ^1^H-NMR spectra were collected on a 500 MHz Inova (Varian Inc., Palo Alto, CA) spectrometer equipped with a 5 mm HCN Z-gradient pulsed-field gradient (PFG) room-temperature probe. ^1^H-NMR spectra were acquired at 25°C using the first transient of the NOESY-presaturation pulse sequence, which was chosen for its high degree of quantitative accuracy. Spectra were collected with 256 transients using a 4s acquisition time and a 1s recycle delay.

### NMR Compound Identification and Quantification

All FIDs (free induction decays) were zero-filled to 64k data points and subjected to line broadening of 0.5 Hz. The singlet produced by the DSS methyl groups was used as an internal standard for chemical shift referencing (set to 0 ppm) and for quantification. All ^1^H-NMR spectra were processed and analyzed using the Chenomx NMR Suite Professional software package version 6.0 (Chenomx Inc., Edmonton, AB). The Chenomx NMR Suite software allows for qualitative and quantitative analysis of an NMR spectrum by manually fitting spectral signatures from an internal database of reference spectra to the full NMR spectrum [56]. Specifically, the spectral fitting for each metabolite was done using the standard Chenomx 500 MHz metabolite library. Typically 90% of all visible peaks were assigned to a compound and more than 90% of the spectral area could be routinely fit using the Chenomx spectral analysis software. Most of the visible peaks are annotated with a compound name. It has been previously shown that this fitting procedure provides absolute concentration accuracies of 90% or better. Each spectrum was processed and analyzed by at least two NMR spectroscopists to minimize compound misidentification and misquantification. We used sample spiking to confirm the identities of assigned compound. The confirmations of the spiking with standard and original peaks in experiments for those unexpected metabolites (i.e. phenylacetate and N-carbamoyl-beta-alanine) and for those hard to assign (i.e. malonic acid and oxypurinol) are shown in **Figure S12** **(A–D)**. Sample spiking involves the addition of 20–200 µM of the suspected compound to selected samples and testing whether the relative NMR signal intensity changed as expected.

### Data Processing for Statistical Analysis

To visualize the compounds, and compare the metabolite changes (composition and concentrations) of all cold perturbation treatments with the control (N_33_ cells grown at 21°C =  GT21 or T0), several multivariate and univariate analytical methods were applied. The water and lipid soluble compound concentration data tables were arranged with samples in column and compounds in rows. Data tables were formatted as comma separated values (.csv). Data tables with three growing temperatures (GT21, GT4, GT10) and 6 time points (T0, T1, T2, T3, T8, T4, T5) were uploaded to the MetaboAnalyst 2.0 server (http://www.metaboanalyst.ca) [57] and analysed separately. In both groups of data metabolite, data were unpaired and analysed using multivariate and univariate methods. To reduce any possible variance and to improve the performance for downstream statistical analysis, metabolites data generated by GC-MS and NMR were normalized using MetaboAnalyst’s normalization protocols [57]. For multivariate analysis (PCA and PLS-DA), Row-wise normalization was performed for all metabolite data by comparing the samples with a pooled average of reference samples (cells grown at 21°C) to make each sample comparable to one another. To make the metabolite concentration values more comparable among different compounds, several different types of column-wise normalization were performed. The fractions of fatty acids (from total, neutral, phospho-, and glyco- lipids) were log transformed and analysed individually. The fatty acids data were also normalized using auto scaling (mean-centered and divided by the standard deviation of each variable). Same normalization procedures were performed for NMR data. Univariate analysis was applied to calculate the fold change (FC), volcano plots, and statistical significance (t-test and one way ANOVA) assessed. Since FC is calculated as the ratio between two group means (sample over control), the column-wise normalization (i.e. log transformation/mean-centering) will significantly change the absolute values, thus in univariate analysis data were used before column normalization [58]. To assess the degree of metabolite concentration changes, heat maps and hierarchical clustering were performed using the MetaboAnalyst 2.0 software [57]. Heatmaps were created based on the Pearson distance measure and the Ward clustering algorithm, displayed for top 25 features selected by analysis of variance (ANOVA) using a significance level of *P*≤0.05, and post-hoc analysis of Fisher’s LSD.

## Supporting Information

Figure S1 **Experimental plan used to study metabolomics of cold adaptation of the Arctic** ***Mesorhizobium*** **N_33_.** Bacteria were cultivated in yeast mannitol broth (YMB) medium at a constant temperature of 21°C (GT21), 4°C (GT4) or 10°C (GT10). For the effect of time of exposure to cold, N_33_ cells were grown at 21°C (T0) and then exposed to 4°C for: 2 min (T1), 4 min (T2), 8 min (T3), 60 min (T4) and 240 min (T5). Each treatment is color coded, and the color codes are used in all figures of this manuscript.
(TIF)

Figure S2 **Heatmap visualization of water soluble metabolites present in** ***Mesorhizobium*** **N_33_ during growth at different temperatures.** GT21 =  growth at 21°C (control); GT4 =  growth at 4°C; GT10 =  growth at 10°C. Data were row-wise normalized by a pooled averaged reference sample (GT21), and were auto scaled and log transformed. Hierarchical clustering was performed based on Pearson’s distance on 29 water soluble metabolites and is shown at the top and side of the panel. Brown and blue colors represent an increase and decrease of a metabolite. The heatmap visualization shows for each growth temperature used a distinct effect Conditions GT21 and GT10 represent close change trends of the water soluble metabolites. However some compounds have shown slightly different levels of accumulations. Metabolites of the cells grown at 4°C (GT4) are clustered in a distinct group far from those of GT10 and GT21. Most influential compounds that were highly accumulated during constant growth at 4°C include isobutyrate, sarcosine, threonine, and valine.
(TIF)

Figure S3 **Heatmap visualization of water soluble metabolites present in** ***Mesorhizobium*** **N_33_ exposed to suboptimal 4°C for various times.** T0 = 21°C (control), T1 = 2 min; T2 = 4 min; T3 = 8 min; T4 = 60 min; T5 = 240 min exposure to 4°C of cells grown at 21°C. Data were row-wise normalized by a pooled averaged reference sample (T0), and were auto scaled and log transformed. Hierarchical clustering was performed based on Pearson’s distance on 29 water soluble metabolites and is shown at the top and side of the panel. Brown and blue colors represent an increase and decrease of a metabolite. The heatmap visualization shows different trends of metabolite changes under each time of exposure to low temperature.
(TIF)

Figure S4 **Heatmap visualization of fatty acids from neutral lipids present in** ***Mesorhizobium*** **N_33_ during growth at different temperatures.** GT21 =  growth at 21°C (control); GT4 =  growth at 4°C; GT10 =  growth at 10°C. Data were row-wise normalized by a pooled averaged reference samples (GT21), and were auto scaled and log transformed. Hierarchical clustering was performed based on Pearson’s distance on 16 fatty acids from neutral lipids and is shown at the top and side of the panel. Brown and blue colors represent an increase and decrease of a metabolite. The fatty acids were clustered in 5 groups. Conditions GT21 and GT10 represent a close change trends in fatty acids. Metabolites of cells grown at the 4°C (GT4) are clustered in distinct group and far from those of GT10 and GT21.
(TIF)

Figure S5 **Heatmap visualization of fatty acids from neutral lipids present in** ***Mesorhizobium*** **N_33_ exposed to suboptimal 4°C for various times.** T0 = 21°C (control), T1 = 2 min; T2 = 4 min; T3 = 8 min; T4 = 60 min; T5 = 240 min exposure to 4°C of cells grown at 21°C. Data were row-wise normalized by a pooled averaged reference sample (T0), and were auto scaled and log transformed. Hierarchical clustering was performed based on Pearson’s distance on 17 fatty acids from neutral lipids and is shown at the top and side of the panel. Brown and blue colors represent an increase and decrease of a metabolite. The heatmap visualization shows different trends of metabolite changes under each time of exposure to low temperature.
(TIF)

Figure S6 **Heatmap visualization of fatty acids from phospholipids present in** ***Mesorhizobium*** **N_33_ during growth at different temperatures.** GT21 =  growth at 21°C (control); GT4 =  growth at 4°C; GT10 =  growth at 10°C. Data were, row-wise normalized by a pooled averaged reference samples (GT21), and were auto scaled and log transformed. Hierarchical clustering was performed based on Pearson’s distance on 17 fatty acids from phospholipids and is shown at the top and side of the panel. Brown and blue colors represent an increase and decrease of a metabolite. The fatty acids were grouped in 2 main clusters and 4 sub-clusters. Conditions GT10 and GT4 represent a close trends of the fatty acids changes Metabolites of the cells grown at the 21°C (GT21) are clustered in distinct group and far from those GT10 and GT4.
(TIF)

Figure S7 **Heatmap visualization of fatty acids from phospholipids present in** ***Mesorhizobium*** **N_33_ exposed to suboptimal 4°C for various times.** T0 = 21°C (control), T1 = 2 min; T2 = 4 min; T3 = 8 min; T4 = 60 min; T5 = 240 min exposure to 4°C of cells grown at 21°C. Data were row-wise normalized by a pooled averaged reference sample (T0), and were auto scaled and log transformed. Hierarchical clustering was performed based on Pearson’s distance on 17 fatty acids from phospholipids and is shown at the top and side of the panel. Brown and blue colors represent an increase and decrease of a metabolite. The heatmap visualization shows different trends of metabolite changes under each time of exposure to low temperature.
(TIF)

Figure S8 **Heatmap visualization of fatty acids from glycolipids present in** ***Mesorhizobium*** **N_33_ during growth at different temperatures.** GT21 =  growth at 21°C (control); GT4 =  growth at 4°C; GT10 =  growth at 10°C. Data were row-wise normalized by a pooled averaged reference samples (GT21), and were auto scaled and log transformed. Hierarchical clustering was performed based on Pearson’s distance on 17 fatty acids from glycolipids and is shown at the top and side of the panel. Brown and blue colors represent an increase and decrease of a metabolite. The heatmap visualization shows different trends of the metabolite changes at 21°C (GT21), 10°C (GT10) and 4°C (GT4). The fatty acids were grouped in 3 main clusters and 5 sub-clusters. Conditions GT4 represents distinct trends of metabolite changes compared to metabolites of the cells grown at 21°C, whereas cells grown at 10°C (GT10) represents an intermediate levels of metabolite changes.
(TIF)

Figure S9 **Heatmap visualization of fatty acids from glycolipids present in** ***Mesorhizobium*** **N_33_ exposed to suboptimal 4°C for various times.** T0 = 21°C (control), T1 = 2 min; T2 = 4 min; T3 = 8 min; T4 = 60 min; T5 = 240 min exposure to 4°C of cells grown at 21°C. Data were row-wise normalized by a pooled averaged reference sample (T0), and were auto scaled and log transformed. Hierarchical clustering was performed based on Pearson’s distance on 17 fatty acids from glycolipids and is shown at the top and side of the panel. Brown and blue colors represent an increase and decrease of a metabolite. The heatmap visualization shows different trends of metabolite changes under each time of exposure to low temperature.
(TIF)

Figure S10 **Heatmap visualization of fatty acids from total lipids present in** ***Mesorhizobium*** **N_33_ during growth at different temperatures.** GT21 =  growth at 21°C (control); GT4 =  growth at 4°C; GT10 =  growth at 10°C. Data were row-wise normalized by a pooled averaged reference samples (GT21), and were auto scaled and log transformed. Hierarchical clustering was performed based on Pearson’s distance on 13 fatty acids and is shown at the top and side of the panel. Brown and blue colors represent an increase and decrease of a metabolite. The heatmap visualization shows distinct trends of the metabolite changes at 21°C (GT21), 10°C (GT10) and 4°C (GT4). The fatty acids were grouped in 3 main clusters and 8 sub-clusters in Heatmaps. Conditions GT4 represents distinct trends of metabolite changes compared to the metabolites of the cells grown at 21°C and 10°C. Out of 13 fatty acids of total lipids, at least 6 fatty acids showed accumulation at 4°C and 7 compounds showed down regulations.
(TIF)

Figure S11 **Heatmap visualization of fatty acids from total lipids present in** ***Mesorhizobium*** **N_33_ exposed to suboptimal 4°C for various times.** T0 = 21°C (control), T1 = 2 min; T2 = 4 min; T3 = 8 min; T4 = 60 min; T5 = 240 min exposure to 4°C of cells grown at 21°C. Data were row-wise normalized by a pooled averaged reference sample (T0), and were auto scaled and log transformed. Hierarchical clustering was performed based on Pearson’s distance on 13 fatty acids from total lipids and is shown at the top and side of the panel. Brown and blue colors represent an increase and decrease of a metabolite. The heatmap visualization shows different trends of metabolite changes under each time exposure at low temperature.
(TIF)

Figure S12 **Confirmations by spiking with standard and original peaks of unexpected metabolites.** (**A**) Malonic acid, (**B**) Oxypurinol, (**C**) Phenylacetate, (**D**) N-Carbamoyl-beta-alanine.
(TIF)

Table S1 **Low temperature effects on the concentration (µM) of 29 water-soluble metabolites determined by NMR in Arctic** ***Mesorhizobium*** **N33.**
(DOCX)

Table S2 **Low temperature effects on the fatty acid composition of total lipids determined by GC-MS in Arctic** ***Mesorhizobium*** **N33 (expressed as mole % of total fatty acids).**
(DOCX)

Table S3 **Low temperature effects on the fatty acid composition of neutral lipids determined by GC-MS in arctic** ***Mesorhizobium*** **strain N33 (expressed as mole % of total neutral fatty acids).**
(DOCX)

Table S4 **Low temperature effects on the fatty acid composition of glycolipids determined by GC-MS in arctic** ***Mesorhizobium*** **strain N33 (expressed as mole % of total glycolipids).**
(DOCX)

Table S5 **Low temperature effects on the fatty acid composition of phospholipids determined by GC-MS in arctic** ***Mesorhizobium*** **strain N33 (expressed as mole % of total phospholipids).**
(DOCX)
