# Supplementary figures and images for: Metabolomic Analysis of Cold Acclimation of Arctic Mesorhizobium sp. Strain N33
Source: PLoS One. 2013 Dec 30;8(12):e84801. doi: 10.1371/journal.pone.0084801 (PMC3875568; doi:10.1371/journal.pone.0084801)

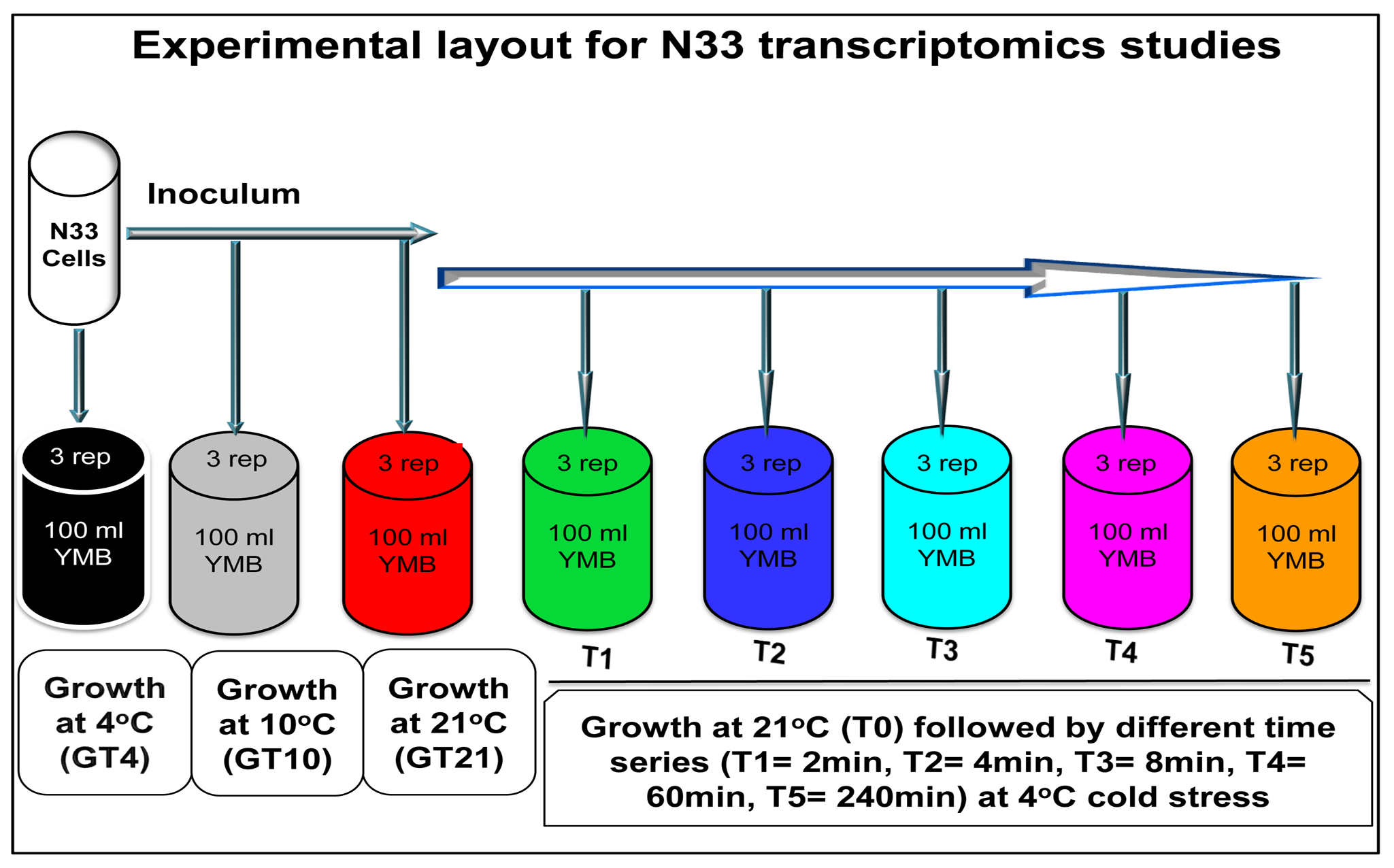

Supplement: Figure S1 — Experimental plan used to study metabolomics of cold adaptation of the Arctic Mesorhizobium N33. Bacteria were cultivated in yeast mannitol broth (YMB) medium at a constant temperature of 21°C (GT21), 4°C (GT4) or 10°C (GT10). For the effect of time of exposure to cold, N33 cells were grown at 21°C (T0) and then exposed to 4°C for: 2 min (T1), 4 min (T2), 8 min (T3), 60 min (T4) and 240 min (T5). Each treatment is color coded, and the color codes are used in all figures of this manuscript. (TIF) [file pone.0084801.s001.tif]

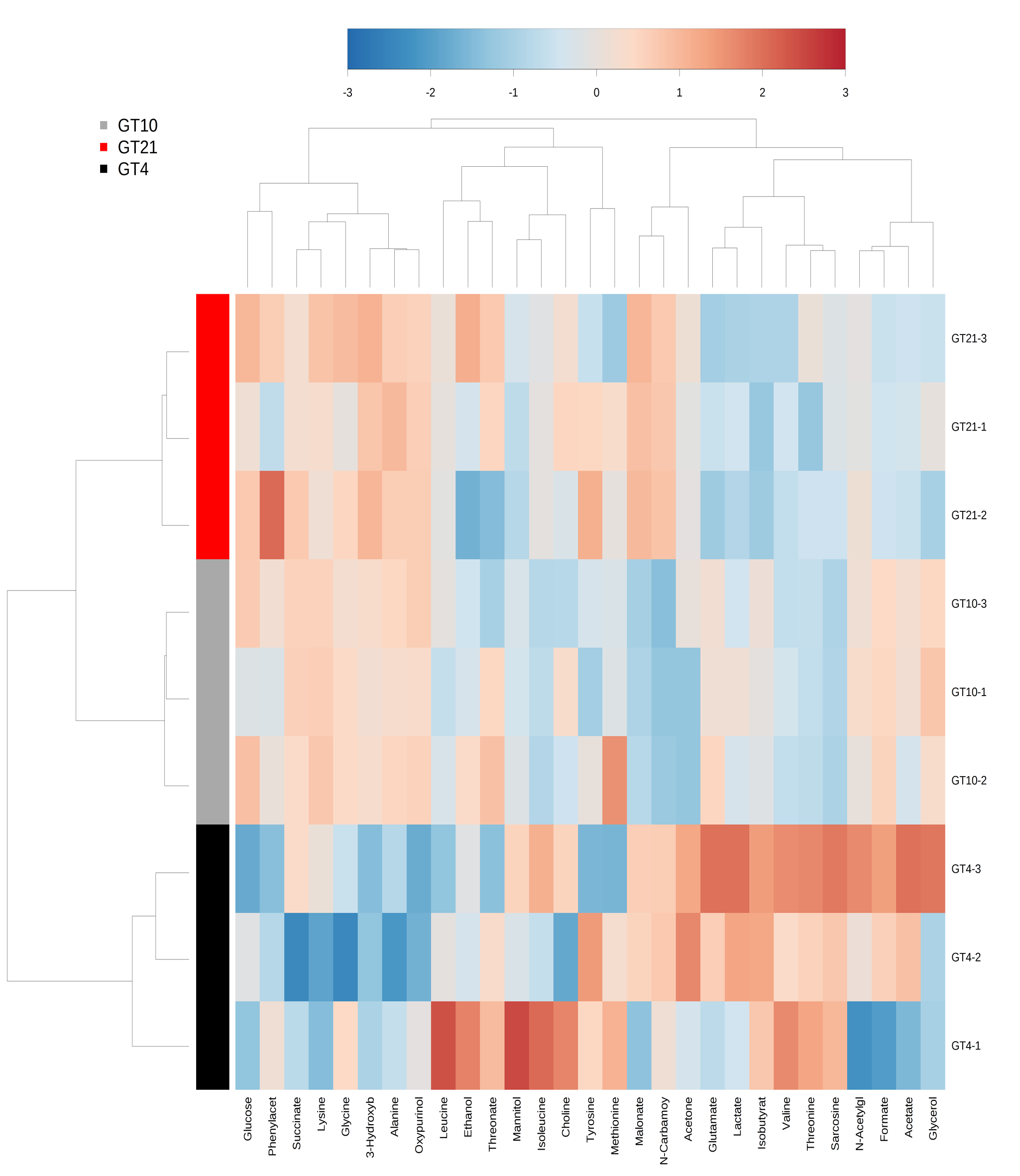

Supplement: Figure S2 — Heatmap visualization of water soluble metabolites present in Mesorhizobium N33 during growth at different temperatures. GT21 = growth at 21°C (control); GT4 = growth at 4°C; GT10 = growth at 10°C. Data were row-wise normalized by a pooled averaged reference sample (GT21), and were auto scaled and log transformed. Hierarchical clustering was performed based on Pearson’s distance on 29 water soluble metabolites and is shown at the top and side of the panel. Brown and blue colors represent an increase and decrease of a metabolite. The heatmap visualization shows for each growth temperature used a distinct effect Conditions GT21 and GT10 represent close change trends of the water soluble metabolites. However some compounds have shown slightly different levels of accumulations. Metabolites of the cells grown at 4°C (GT4) are clustered in a distinct group far from those of GT10 and GT21. Most influential compounds that were highly accumulated during constant growth at 4°C include isobutyrate, sarcosine, threonine, and valine. (TIF) [file pone.0084801.s002.tif]

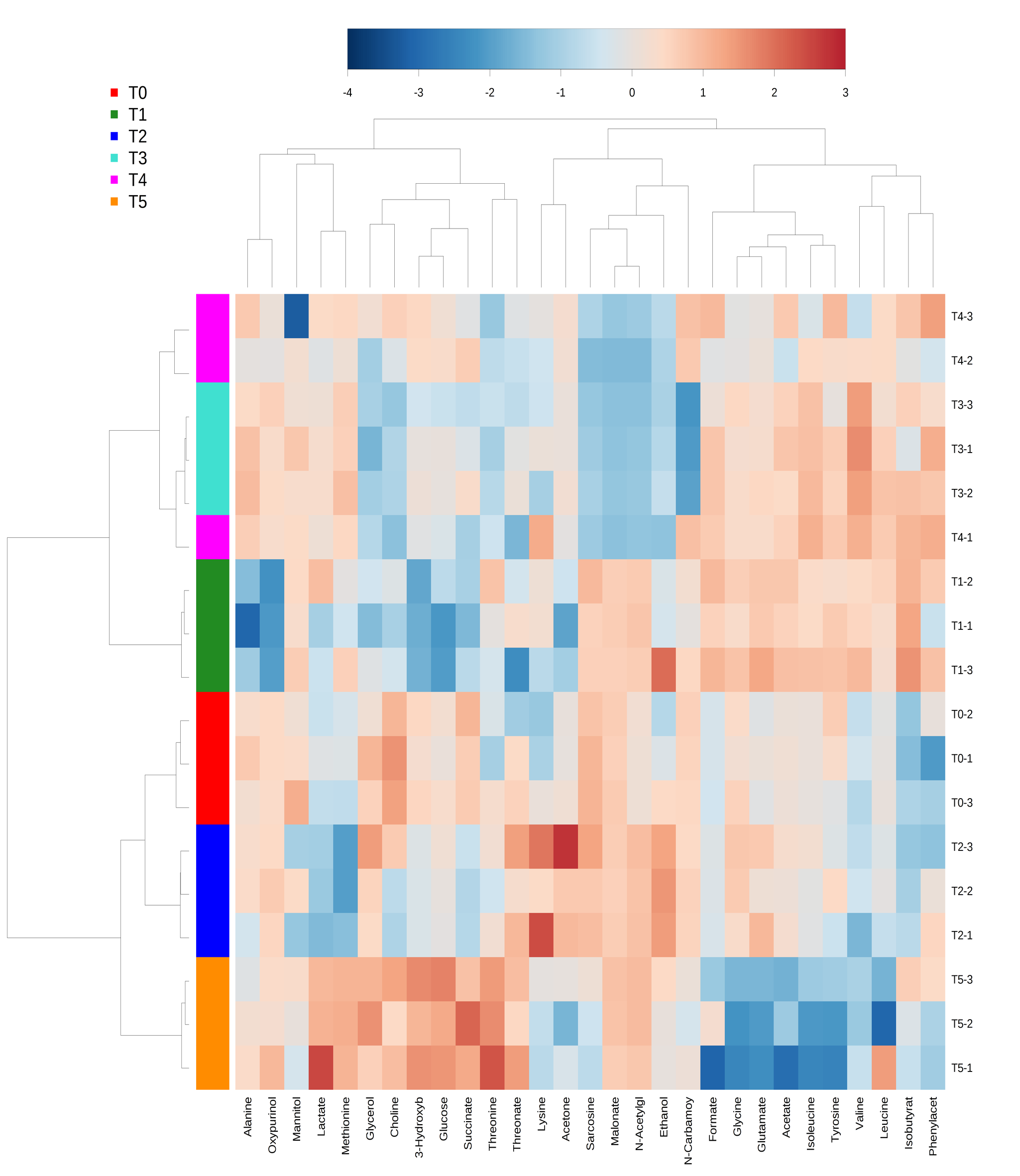

Supplement: Figure S3 — Heatmap visualization of water soluble metabolites present in Mesorhizobium N33 exposed to suboptimal 4°C for various times. T0 = 21°C (control), T1 = 2 min; T2 = 4 min; T3 = 8 min; T4 = 60 min; T5 = 240 min exposure to 4°C of cells grown at 21°C. Data were row-wise normalized by a pooled averaged reference sample (T0), and were auto scaled and log transformed. Hierarchical clustering was performed based on Pearson’s distance on 29 water soluble metabolites and is shown at the top and side of the panel. Brown and blue colors represent an increase and decrease of a metabolite. The heatmap visualization shows different trends of metabolite changes under each time of exposure to low temperature. (TIF) [file pone.0084801.s003.tif]

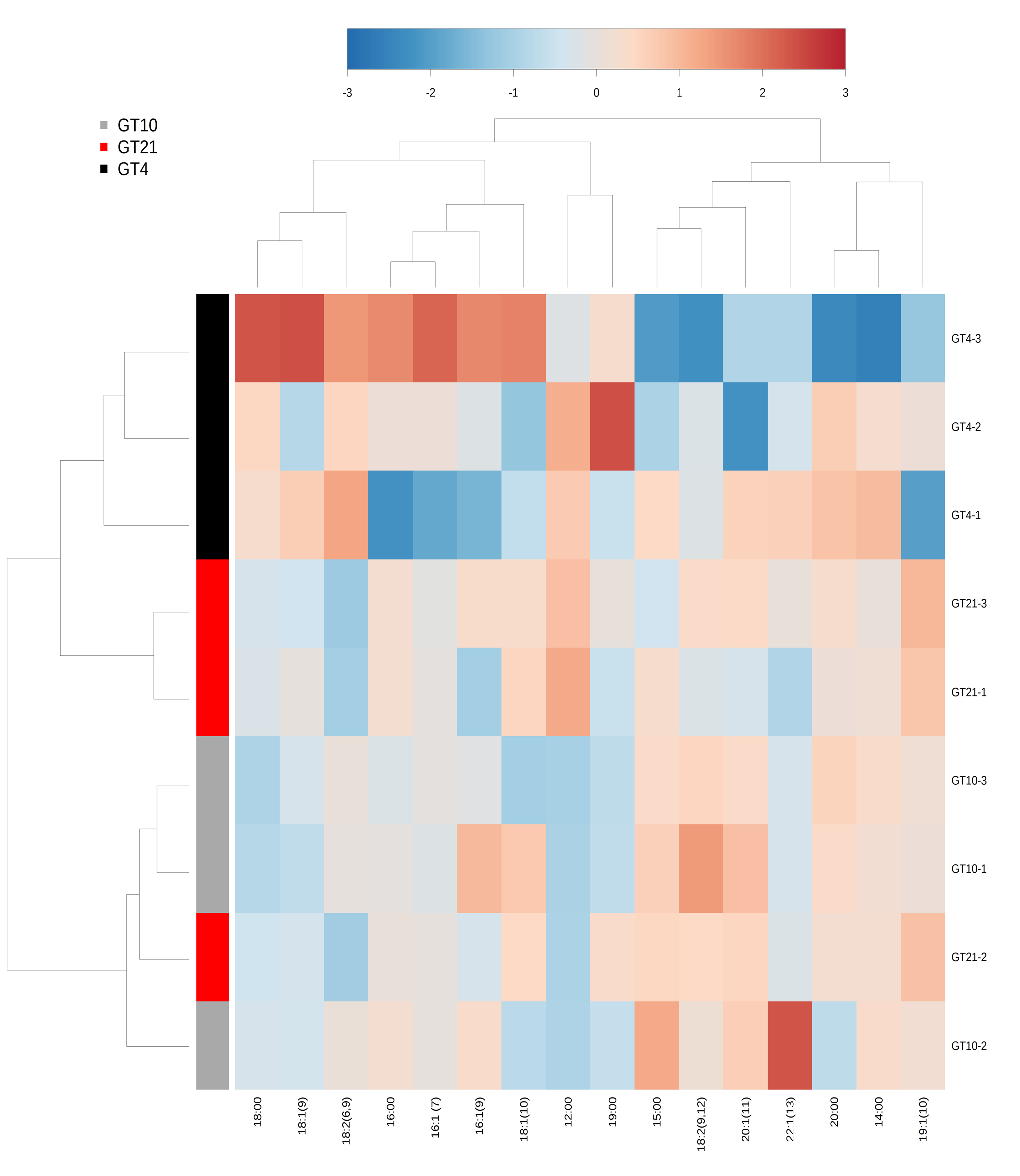

Supplement: Figure S4 — Heatmap visualization of fatty acids from neutral lipids present in Mesorhizobium N33 during growth at different temperatures. GT21 = growth at 21°C (control); GT4 = growth at 4°C; GT10 = growth at 10°C. Data were row-wise normalized by a pooled averaged reference samples (GT21), and were auto scaled and log transformed. Hierarchical clustering was performed based on Pearson’s distance on 16 fatty acids from neutral lipids and is shown at the top and side of the panel. Brown and blue colors represent an increase and decrease of a metabolite. The fatty acids were clustered in 5 groups. Conditions GT21 and GT10 represent a close change trends in fatty acids. Metabolites of cells grown at the 4°C (GT4) are clustered in distinct group and far from those of GT10 and GT21. (TIF) [file pone.0084801.s004.tif]

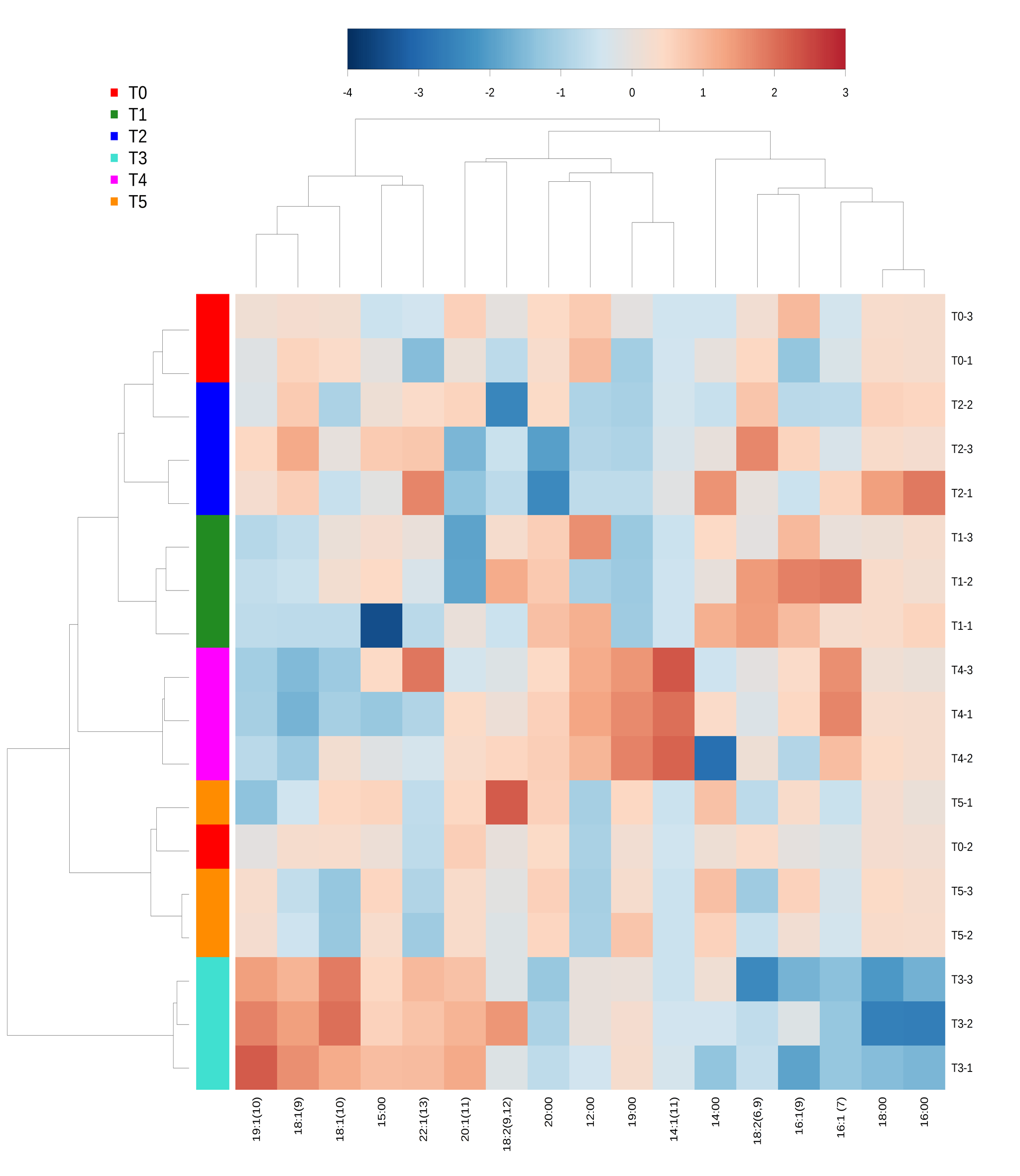

Supplement: Figure S5 — Heatmap visualization of fatty acids from neutral lipids present in Mesorhizobium N33 exposed to suboptimal 4°C for various times. T0 = 21°C (control), T1 = 2 min; T2 = 4 min; T3 = 8 min; T4 = 60 min; T5 = 240 min exposure to 4°C of cells grown at 21°C. Data were row-wise normalized by a pooled averaged reference sample (T0), and were auto scaled and log transformed. Hierarchical clustering was performed based on Pearson’s distance on 17 fatty acids from neutral lipids and is shown at the top and side of the panel. Brown and blue colors represent an increase and decrease of a metabolite. The heatmap visualization shows different trends of metabolite changes under each time of exposure to low temperature. (TIF) [file pone.0084801.s005.tif]

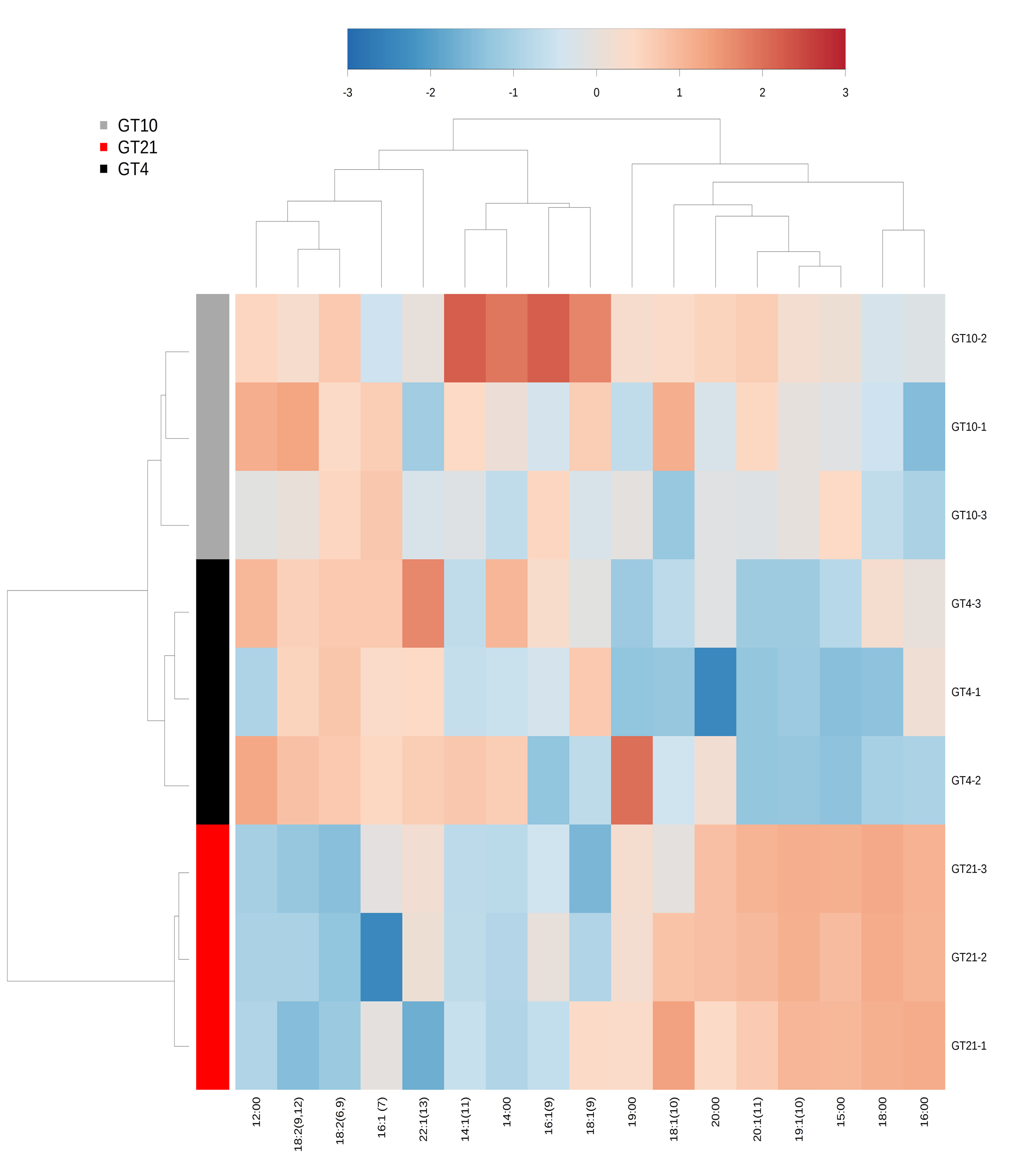

Supplement: Figure S6 — Heatmap visualization of fatty acids from phospholipids present in Mesorhizobium N33 during growth at different temperatures. GT21 = growth at 21°C (control); GT4 = growth at 4°C; GT10 = growth at 10°C. Data were, row-wise normalized by a pooled averaged reference samples (GT21), and were auto scaled and log transformed. Hierarchical clustering was performed based on Pearson’s distance on 17 fatty acids from phospholipids and is shown at the top and side of the panel. Brown and blue colors represent an increase and decrease of a metabolite. The fatty acids were grouped in 2 main clusters and 4 sub-clusters. Conditions GT10 and GT4 represent a close trends of the fatty acids changes Metabolites of the cells grown at the 21°C (GT21) are clustered in distinct group and far from those GT10 and GT4. (TIF) [file pone.0084801.s006.tif]

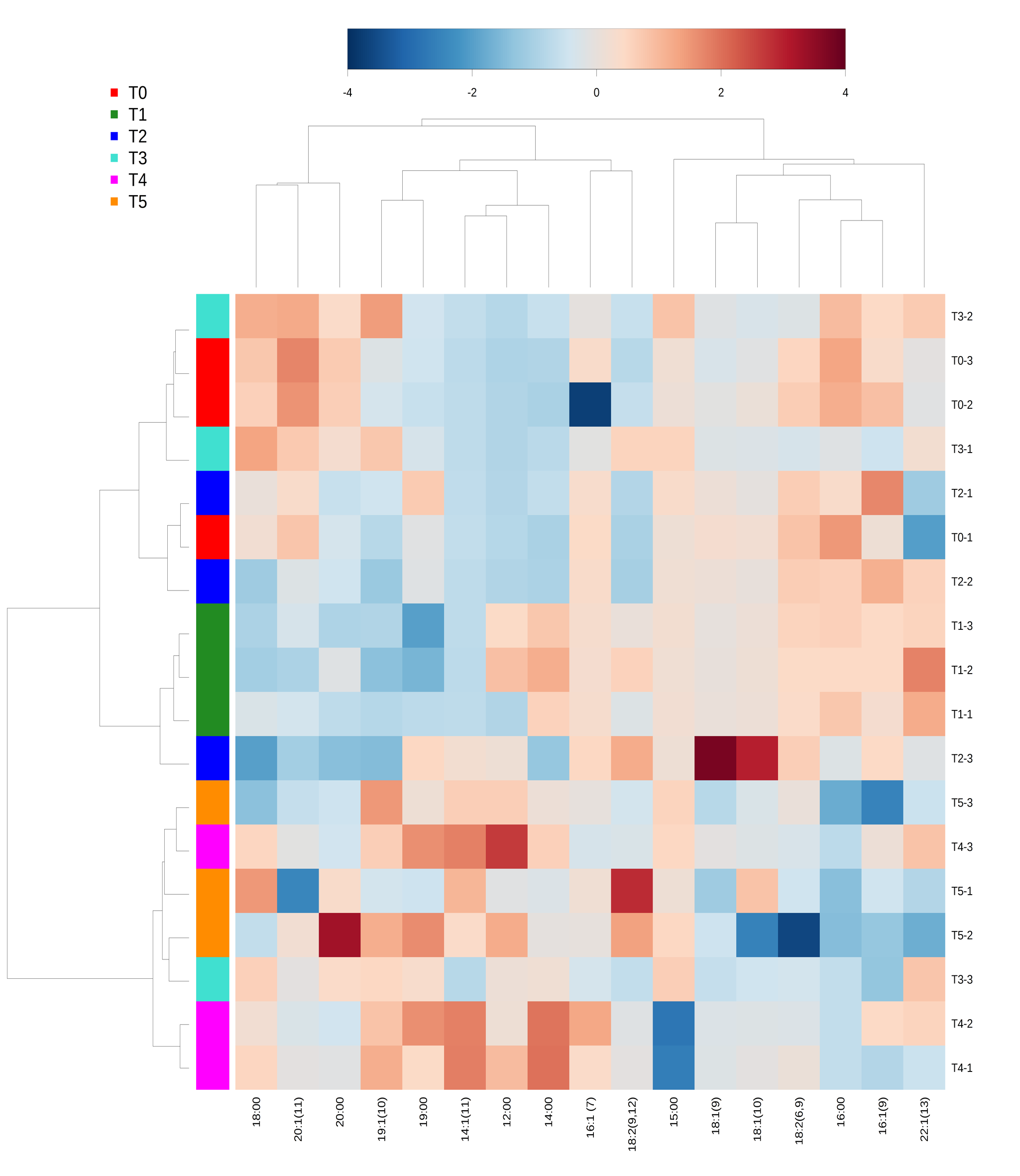

Supplement: Figure S7 — Heatmap visualization of fatty acids from phospholipids present in Mesorhizobium N33 exposed to suboptimal 4°C for various times. T0 = 21°C (control), T1 = 2 min; T2 = 4 min; T3 = 8 min; T4 = 60 min; T5 = 240 min exposure to 4°C of cells grown at 21°C. Data were row-wise normalized by a pooled averaged reference sample (T0), and were auto scaled and log transformed. Hierarchical clustering was performed based on Pearson’s distance on 17 fatty acids from phospholipids and is shown at the top and side of the panel. Brown and blue colors represent an increase and decrease of a metabolite. The heatmap visualization shows different trends of metabolite changes under each time of exposure to low temperature. (TIF) [file pone.0084801.s007.tif]

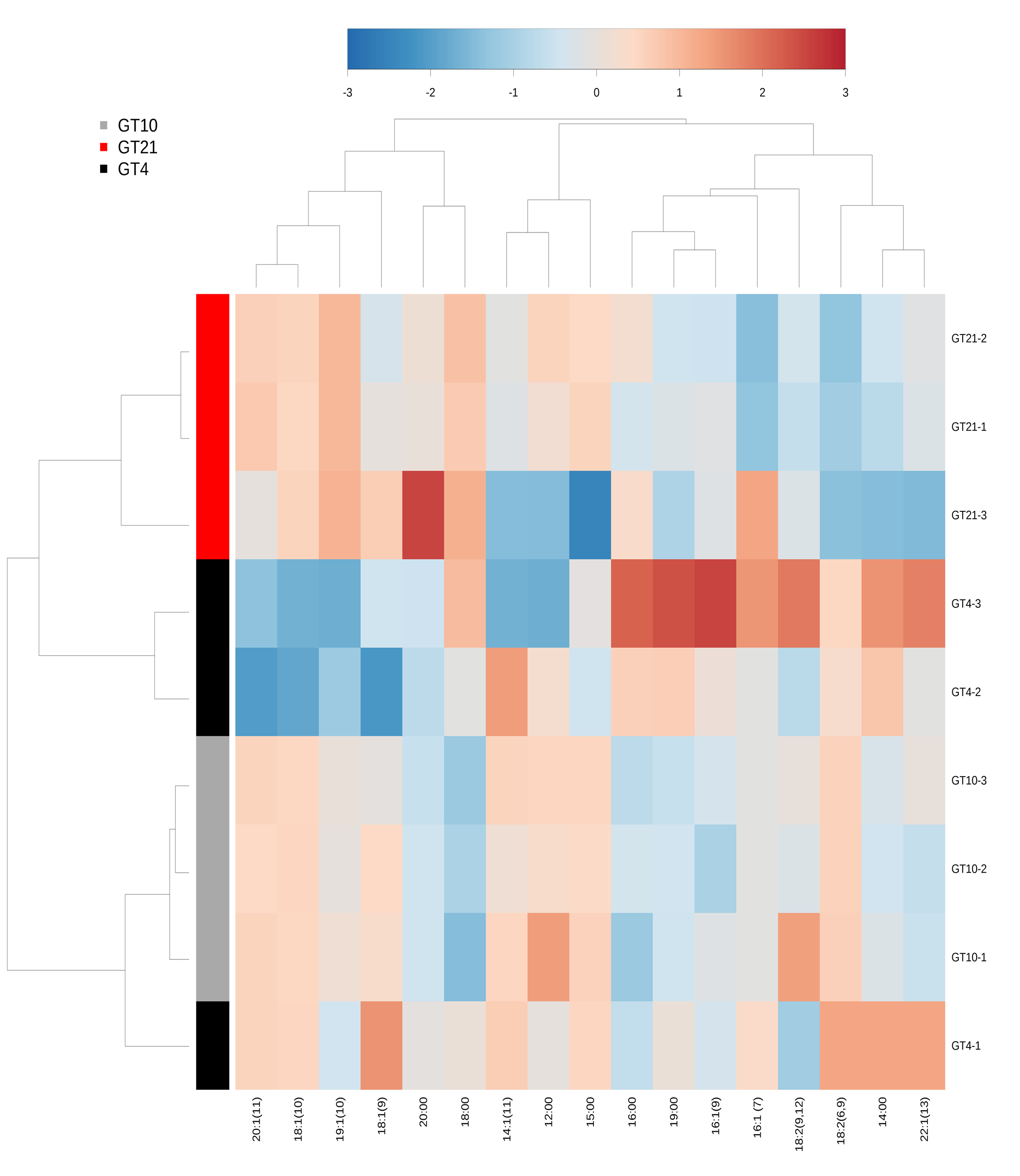

Supplement: Figure S8 — Heatmap visualization of fatty acids from glycolipids present in Mesorhizobium N33 during growth at different temperatures. GT21 = growth at 21°C (control); GT4 = growth at 4°C; GT10 = growth at 10°C. Data were row-wise normalized by a pooled averaged reference samples (GT21), and were auto scaled and log transformed. Hierarchical clustering was performed based on Pearson’s distance on 17 fatty acids from glycolipids and is shown at the top and side of the panel. Brown and blue colors represent an increase and decrease of a metabolite. The heatmap visualization shows different trends of the metabolite changes at 21°C (GT21), 10°C (GT10) and 4°C (GT4). The fatty acids were grouped in 3 main clusters and 5 sub-clusters. Conditions GT4 represents distinct trends of metabolite changes compared to metabolites of the cells grown at 21°C, whereas cells grown at 10°C (GT10) represents an intermediate levels of metabolite changes. (TIF) [file pone.0084801.s008.tif]

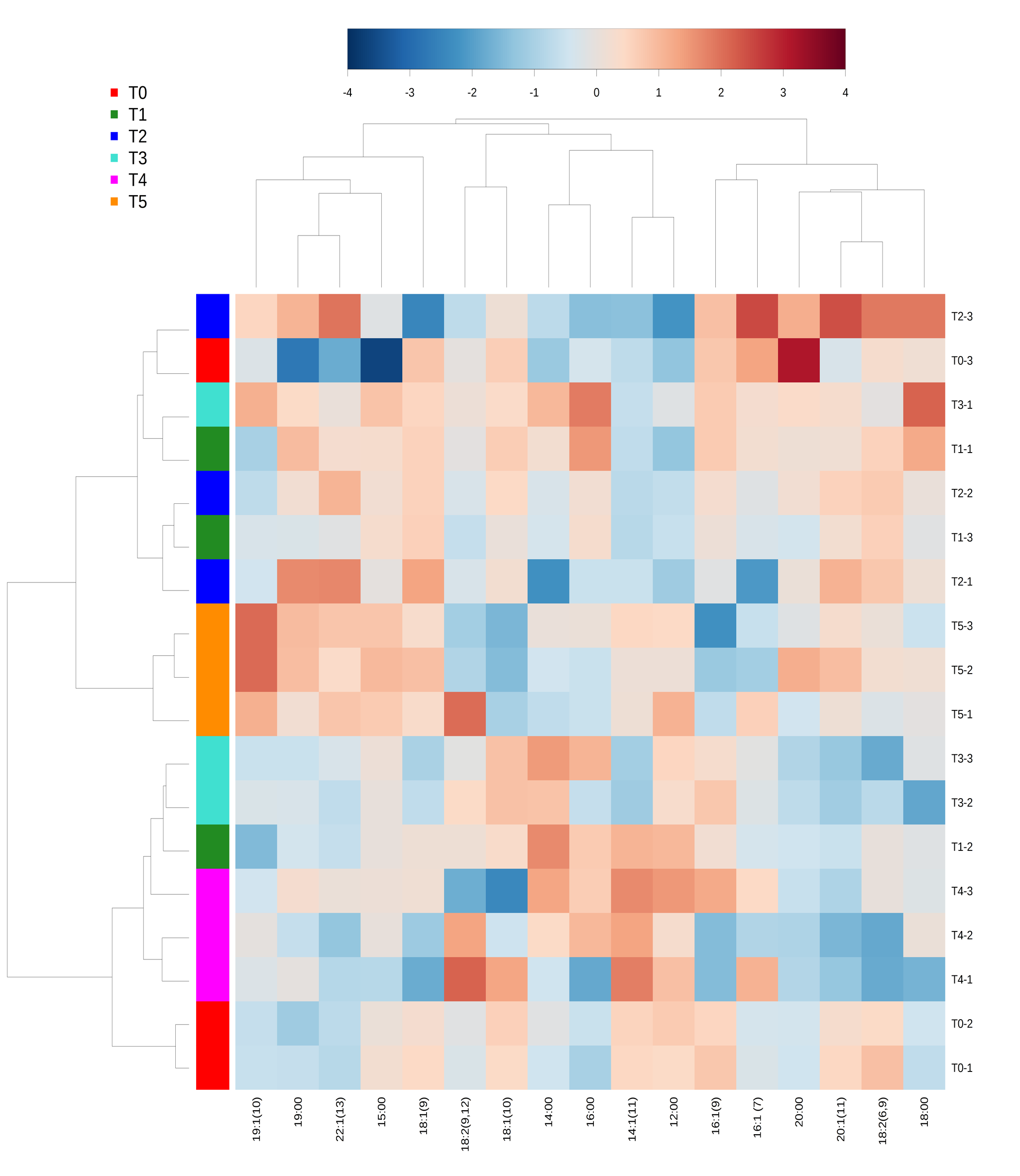

Supplement: Figure S9 — Heatmap visualization of fatty acids from glycolipids present in Mesorhizobium N33 exposed to suboptimal 4°C for various times. T0 = 21°C (control), T1 = 2 min; T2 = 4 min; T3 = 8 min; T4 = 60 min; T5 = 240 min exposure to 4°C of cells grown at 21°C. Data were row-wise normalized by a pooled averaged reference sample (T0), and were auto scaled and log transformed. Hierarchical clustering was performed based on Pearson’s distance on 17 fatty acids from glycolipids and is shown at the top and side of the panel. Brown and blue colors represent an increase and decrease of a metabolite. The heatmap visualization shows different trends of metabolite changes under each time of exposure to low temperature. (TIF) [file pone.0084801.s009.tif]

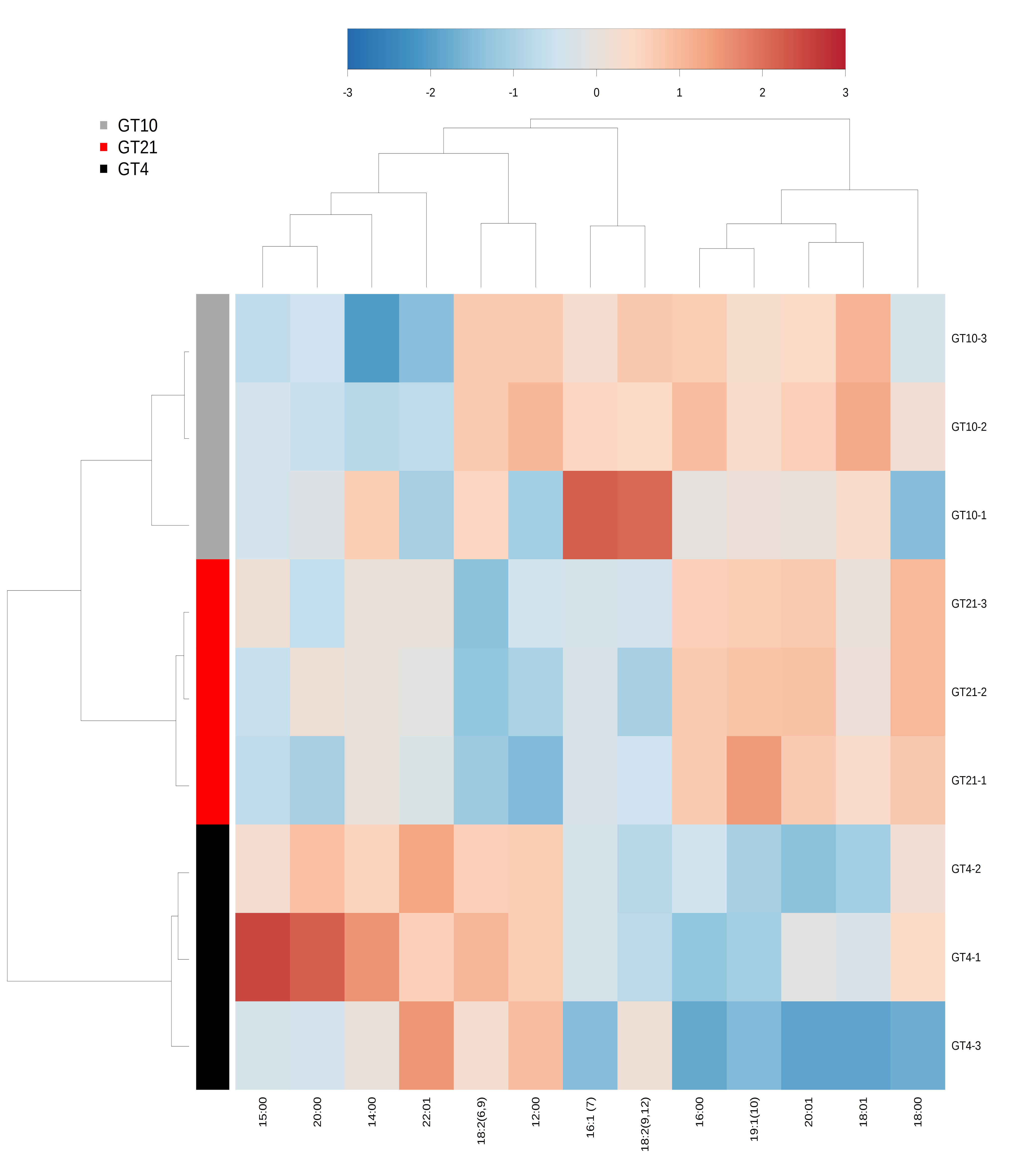

Supplement: Figure S10 — Heatmap visualization of fatty acids from total lipids present in Mesorhizobium N33 during growth at different temperatures. GT21 = growth at 21°C (control); GT4 = growth at 4°C; GT10 = growth at 10°C. Data were row-wise normalized by a pooled averaged reference samples (GT21), and were auto scaled and log transformed. Hierarchical clustering was performed based on Pearson’s distance on 13 fatty acids and is shown at the top and side of the panel. Brown and blue colors represent an increase and decrease of a metabolite. The heatmap visualization shows distinct trends of the metabolite changes at 21°C (GT21), 10°C (GT10) and 4°C (GT4). The fatty acids were grouped in 3 main clusters and 8 sub-clusters in Heatmaps. Conditions GT4 represents distinct trends of metabolite changes compared to the metabolites of the cells grown at 21°C and 10°C. Out of 13 fatty acids of total lipids, at least 6 fatty acids showed accumulation at 4°C and 7 compounds showed down regulations. (TIF) [file pone.0084801.s010.tif]

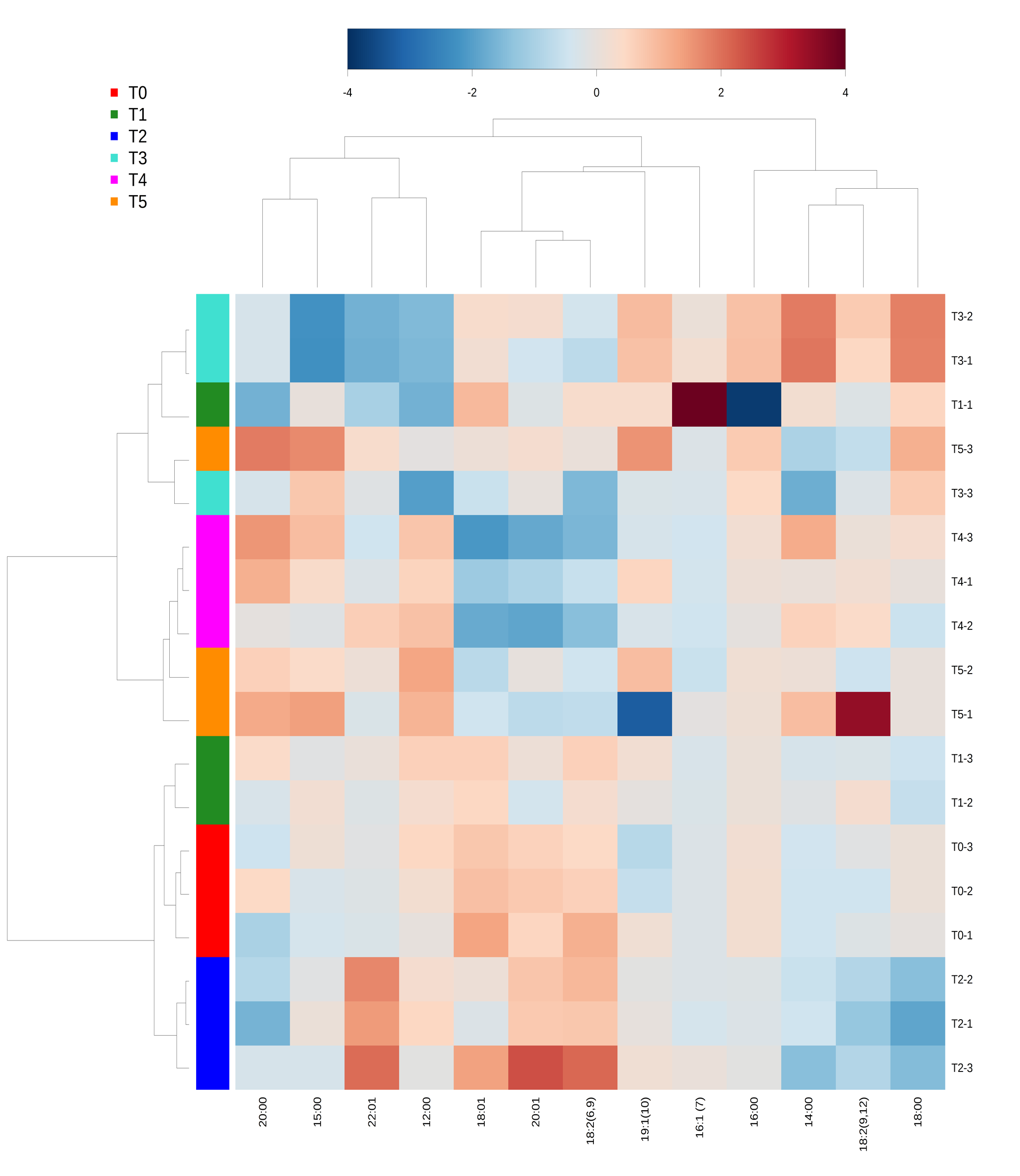

Supplement: Figure S11 — Heatmap visualization of fatty acids from total lipids present in Mesorhizobium N33 exposed to suboptimal 4°C for various times. T0 = 21°C (control), T1 = 2 min; T2 = 4 min; T3 = 8 min; T4 = 60 min; T5 = 240 min exposure to 4°C of cells grown at 21°C. Data were row-wise normalized by a pooled averaged reference sample (T0), and were auto scaled and log transformed. Hierarchical clustering was performed based on Pearson’s distance on 13 fatty acids from total lipids and is shown at the top and side of the panel. Brown and blue colors represent an increase and decrease of a metabolite. The heatmap visualization shows different trends of metabolite changes under each time exposure at low temperature. (TIF) [file pone.0084801.s011.tif]

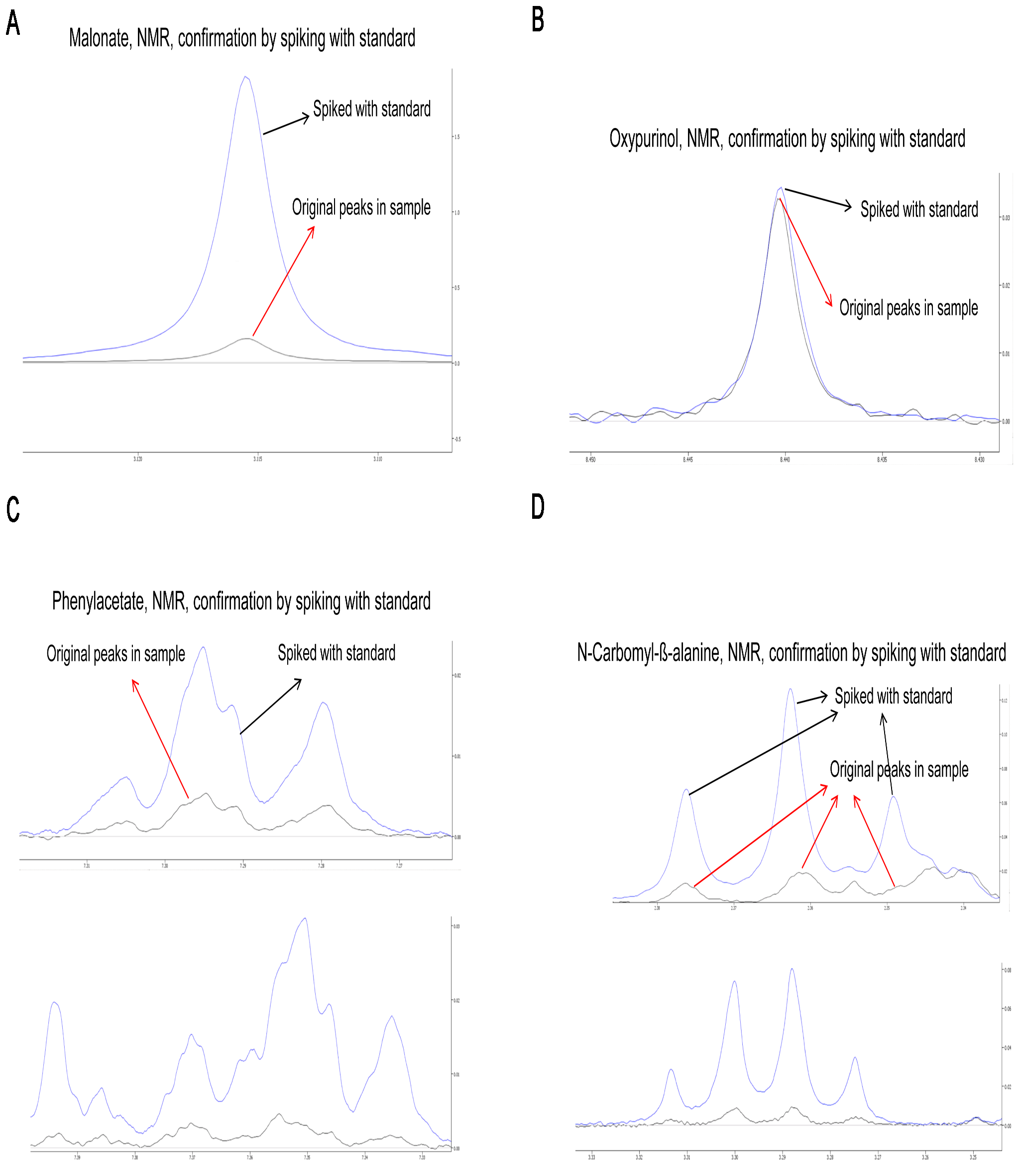

Supplement: Figure S12 — Confirmations by spiking with standard and original peaks of unexpected metabolites. (A) Malonic acid, (B) Oxypurinol, (C) Phenylacetate, (D) N-Carbamoyl-beta-alanine. (TIF) [file pone.0084801.s012.tif]
