# Supplementary material for: Metabolomic Analysis of Cold Acclimation of Arctic Mesorhizobium sp. Strain N33
Source: PLoS One. 2013 Dec 30;8(12):e84801. doi: 10.1371/journal.pone.0084801 (PMC3875568; doi:10.1371/journal.pone.0084801)
Supplement: Table S1 — Low temperature effects on the concentration (µM) of 29 water-soluble metabolites determined by NMR in Arctic Mesorhizobium N33. (DOCX) [file pone.0084801.s013.docx]

**Table S1**. Low temperature effects on the concentration (µM) of 29 water-soluble metabolites determined by NMR in Arctic *Mesorhizobium* N33.

|  |  | GT4 | GT10 | GT21 (T0) | T1 | T2 | T3 | T4 | T5 |
| --- | --- | --- | --- | --- | --- | --- | --- | --- | --- |
| Experiment conditions |  | **Growth at 4^o^C** | **Growth at 10^o^C** | **Growth at 21^o^C** | **Exposed to cold temperature (4 ^o^C) for:** | | | | |
|  |  |  |  |  | **2min** | **4min** | **8min** | **1h** | **4h** |
| Compound Name | **HMDB ID** |  |  |  |  |  |  |  |  |
| 3-Hydroxybutyrate | HMDB00357 | 23.7 ± * | 377.2 ± 111.06 | 559.63 ± 89.86 | ND | 102.23 ± 27.63 | 160.07 ± 68.61 | 450.63 ± 305.72 | 2639.6 ± 1856.86 |
| Acetate | HMDB00042 | 249.03 ± 89.08 | 1145.87 ± 400.72 | 382.33 ± 86.03 | 403.03 ± 90.75 | 352.43 ± 87.2 | 508.6 ± 108.91 | 415.63 ± 105.27 | 35.2 ± 23.9 |
| Acetone | HMDB01659 | 27.77 ± 3.02 | 44.43 ± 41.44 | 30.43 ± 3.1 | 9.45 ± 6.58 | 217.73 ± 282.99 | 28.87 ± 2.29 | 33.1 ± 12.14 | 9.4 ± 4.24 |
| Alanine | HMDB00161 | 209.97 ± 251.06 | 1463.47 ± 351.27 | 743.23 ± 205.23 | 235 ± 89.25 | 574.87 ± 176.85 | 735.07 ± 41.66 | 716.33 ± 136.51 | 290.2 ± 145.2 |
| Choline | HMDB00097 | 62.67 ± 84.48 | 138.9 ± 35.45 | 71 ± 20.05 | 16.97 ± 6.79 | 27.77 ± 20.03 | 17.23 ± 1.35 | 29.2 ± 12.83 | 22.77 ± 8.98 |
| Ethanol | HMDB00108 | 200.7 ± 287.57 | 361 ± 31.84 | 158.53 ± 81.99 | 398.27 ± 529.7 | 800.3 ± 134.12 | 64.6 ± 6.32 | 57.33 ± 23.79 | 85.77 ± 17.4 |
| Formate | HMDB00142 | 134.17 ± 50.64 | 985.13 ± 199.71 | 230.8 ± 52.78 | 340.23 ± 97.16 | 210.33 ± 48.85 | 385.3 ± 62.53 | 394.27 ± 43.94 | 68.7 ± 74.76 |
| Glucose | HMDB00122 | 44.13 ± 40.01 | 480.8 ± 179.39 | 221.83 ± 24.07 | 36.4 ± * | 146.23 ± 63.28 | 108.87 ± 42.64 | 205.37 ± 151.84 | 870.3 ± 353.96 |
| Glutamate | HMDB03339 | 84.2 ± 40.13 | 355.33 ± 59.62 | 82.63 ± 29.28 | 205.17 ± 54.92 | 194.43 ± 88.66 | 131.13 ± 8.33 | 111 ± 42.51 | ND |
| Glycerol | HMDB00131 | 86.47 ± 58.6 | 488.67 ± 130.02 | 136.53 ± 50.43 | 47.47 ± 17.85 | 130.23 ± 62.83 | 49.23 ± 10.6 | 72.33 ± 8.55 | 70.27 ± 29.64 |
| Glycine | HMDB00123 | 47.93 ± 65.12 | 210.9 ± 40.06 | 95.07 ± 5.81 | 80.87 ± 22.58 | 109.57 ± 43.44 | 86.97 ± 20.07 | 67.77 ± 25.71 | ND |
| Isobutyrate | HMDB01873 | 16.4 ± 10.07 | 18.4 ± 7.53 | ND | 11.53 ± 0.67 | ND | 8.47 ± 3.2 | 10.07 ± 3.34 | ND |
| Isoleucine | HMDB00172 | 29.83 ± 38.89 | 45.4 ± 10.73 | 25.63 ± 5.64 | 24.63 ± 3 | 21.7 ± 7.84 | 44.13 ± 3.79 | 38.6 ± 21.32 | ND |
| Lactate | HMDB00190 | 93.03 ± 43.81 | 168.6 ± 58.08 | 44.33 ± 20.48 | 58.17 ± 71.37 | 15.8 ± 6.45 | 78.93 ± 4.65 | 73.17 ± 1.05 | 229.23 ± 276.65 |
| Leucine | HMDB00687 | 23.23 ± 32.8 | 48.3 ± 14.31 | 22.53 ± 4.26 | 18.2 ± 5.44 | 17.3 ± 5.05 | 27.03 ± 0.68 | 29.27 ± 8.95 | 26.3 ± * |
| Lysine | HMDB00182 | 15.15 ± 3.61 | 186.17 ± 30.95 | 66.13 ± 8.58 | 58.47 ± 22.64 | 158.7 ± 60.9 | 68.9 ± 20.2 | 101.83 ± 40.38 | 29.7 ± 9.26 |
| Malonate | HMDB00691 | 56.9 ± 38.98 | 56 ± 1.59 | 620.63 ± 58.93 | 384.8 ± 94.36 | 508.43 ± 104.24 | ND | ND | 349.1 ± 107.76 |
| Mannitol | HMDB00765 | 7186.53 ± 11858.17 | 1859.8 ± 424.86 | 564.83 ± 76.42 | 353.57 ± 69.84 | 276.03 ± 152.26 | 478.63 ± 91.5 | 392.77 ± 302.82 | 168.2 ± 46.49 |
| Methionine | HMDB00696 | 25.8 ± 31.18 | 94.8 ± 10.51 | 37.13 ± 12.42 | 31.4 ± 8.4 | 12.1 ± 1.23 | 66.8 ± 4.69 | 57.7 ± 11.92 | 40.17 ± 17.24 |
| N-Acetylglycine | HMDB00532 | 17.17 ± 9.72 | 104.9 ± 28.81 | 40.23 ± 8.79 | 75.47 ± 18.38 | 127.43 ± 39.3 | ND | ND | 63.77 ± 20.89 |
| N-Carbamoyl-β-alanine | HMDB00026 | 23.07 ± 6.56 | ND | 96.1 ± 23.85 | 40.9 ± 12.4 | 77.4 ± 11.09 | ND | 134.97 ± 31.35 | 18.27 ± 10.78 |
| Oxypurinol | HMDB00786 | 324.9 ± * | 2165.37 ± 1056.72 | 1081.8 ± 300.73 | ND | 1203.17 ± 369.37 | 1049.93 ± 431.6 | 536.17 ± 248.54 | 907.8 ± 1103.11 |
| Phenylacetate | HMDB00209 | 16.5 ± * | 28.3 ± 8 | 15.97 ± 7.65 | 18.57 ± 7.18 | 18.03 ± 3.95 | 29.87 ± 4.99 | 31.37 ± 5.35 | 7.37 ± 1.12 |
|  |  |  |  |  |  |  |  |  |  |
| Sarcosine | HMDB00271 | 66.73 ± 54.57 | 34.3 ± 8.4 | 26.83 ± 6.13 | 13.8 ± 6.8 | 24.77 ± 13.19 | ND | ND | ND |
| Succinate | HMDB00254 | 56.33 ± 58.21 | 321.2 ± 81.86 | 133.3 ± 29.69 | 39.23 ± 10.17 | 59.73 ± 16.39 | 79.8 ± 6.88 | 96.37 ± 62.08 | 75.87 ± 39.6 |
| Threonate | HMDB00943 | 62.8 ± 81.95 | 196.43 ± 41.07 | 82.7 ± 37.19 | 40.93 ± 26.35 | 117.1 ± 53.41 | 62.53 ± 7.24 | 52.17 ± 22.74 | 60.5 ± 39.13 |
| Threonine | HMDB00167 | 61.5 ± 64.71 | 59.77 ± 15.36 | 27.77 ± 7.55 | 28.57 ± 19.6 | 29.87 ± 10.53 | 18.1 ± 4.95 | 20.4 ± 9.25 | 60.93 ± 44.9 |
| Tyrosine | HMDB00158 | 16.57 ± 18.18 | 50.07 ± 13.06 | 29.3 ± 11.48 | 22.37 ± 1.38 | 18.97 ± 7.56 | 27.2 ± 4.69 | 35.27 ± 3.57 | ND |
| Valine | HMDB00883 | 95.67 ± 108.32 | 186.5 ± 43.05 | 78.67 ± 22.11 | 82.7 ± 11.35 | 61.33 ± 22.4 | 161.6 ± 22.79 | 118.7 ± 55.63 | 29.63 ± 15.71 |

*Observed only in one sample

ND: Not detected

Value are means ± standard deviation
