# Supplementary material for: Metabolomic Analysis of Cold Acclimation of Arctic Mesorhizobium sp. Strain N33
Source: PLoS One. 2013 Dec 30;8(12):e84801. doi: 10.1371/journal.pone.0084801 (PMC3875568; doi:10.1371/journal.pone.0084801)
Supplement: Table S2 — Low temperature effects on the fatty acid composition of total lipids determined by GC-MS in Arctic Mesorhizobium N33 (expressed as mole % of total fatty acids). (DOCX) [file pone.0084801.s014.docx]

**Table S2**. Low temperature effects on the fatty acid composition of total lipids determined by GC-MS in Arctic *Mesorhizobium* N33 (expressed as mole % of total fatty acids).

| Experiment conditions | GT4 | GT10 | GT21 (T0) | T1 | T2 | | T3 | | T4 | T5 | |
| --- | --- | --- | --- | --- | --- | --- | --- | --- | --- | --- | --- |
|  | **Growth at 4^o^C** | **Growth at 10^o^C** | **Growth at 21^o^C** | **Exposed to cold temperature (4 ^o^C) for:** | | | | | | | |
|  |  |  |  | **2min** | **4min** | | **8min** | | **1h** | **4h** | |
| Fatty acids from total lipids | | | | | | | | | | |  |
| 12:0 | 0.26 ± 0.09 | 0.11 ± 0.02 | 0.1 ± 0.02 | 0.12 ± 0.02 | | 0.12 ± 0.04 | | ND | 0.2 ± 0.04 | 0.17 ± 0.1 | |
| 14:0 | 1.55 ± 0.36 | 0.46 ± 0.47 | 0.67 ± 0.05 | 0.81 ± 0.04 | | 0.74 ± 0.26 | | 1.15 ± 0.61 | 1.6 ± 0.47 | 1 ± 0.53 | |
| 15:0 | 0.18 ± 0.04 | 0.06 ± 0.02 | 0.08 ± 0.02 | 0.1 ± 0.02 | | 0.11 ± 0.03 | | 0.16 ± * | 0.17 ± 0.05 | 0.18 ± 0.05 | |
| 16:0 | 25.33 ± 6.25 | 19.95 ± 1.52 | 26.17 ± 1.58 | 17.87 ± 14.43 | | 24.03 ± 3.01 | | 35.03 ± 0.75 | 35.08 ± 3.18 | 30.68 ± 0.61 | |
| 16:1 (7) | 1.54 ± 0.24 | 1.35 ± 0.68 | 1.14 ± 0.08 | 10.47 ± 15.99 | | 1.48 ± 0.01 | | 1.28 ± 0.1 | 1.46 ± 0.1 | 1.21 ± 0.35 | |
| 18:0 | 16.22 ± 2.07 | 8.41 ± 0.54 | 13.45 ± 1.16 | 13.09 ± 0.67 | | 10.44 ± 1.5 | | 18.26 ± 0.49 | 19.21 ± 1.89 | 16.09 ± 0.72 | |
| 18:1 | 41.91 ± 5.14 | 61.71 ± 3.51 | 53.93 ± 1.47 | 51 ± 2.07 | | 55.29 ± 4.86 | | 36.22 ± 2.1 | 34.27 ± 3.53 | 38.23 ± 4.34 | |
| 18:2(6,9) | 11.54 ± 3.32 | 6.23 ± 0.13 | 0.47 ± 0.05 | 0.42 ± 0.07 | | 0.75 ± 0.12 | | 0.2 ± 0.02 | 0.28 ± 0.05 | 0.3 ± 0.02 | |
| 18:2(9,12) | 0.25 ± 0.14 | 0.26 ± 0.16 | 0.14 ± 0.02 | 0.18 ± 0.06 | | 0.09 ± 0.01 | | 0.22 ± 0.05 | 0.33 ± 0.07 | 1.66 ± 2.69 | |
| 19:1(10) | 0.32 ± 0.04 | 0.98 ± 0.05 | 3.19 ± 1.46 | 5.38 ± 0.52 | | 5.52 ± 0.49 | | 7.09 ± 2.73 | 6.57 ± 2.67 | 9.74 ± 8.4 | |
| 20:0 | 0.37 ± 0.03 | 0.16 ± 0.04 | 0.19 ± 0.03 | 0.19 ± 0.05 | | 0.21 ± 0.02 | | 0.18 ± 0.04 | 0.34 ± 0.03 | 0.26 ± 0.04 | |
| 20:1 | 0.28 ± 0.03 | 0.25 ± 0.03 | 0.37 ± 0.03 | 0.31 ± 0.04 | | 0.55 ± 0.05 | | 0.29 ± 0.07 | 0.3 ± 0.02 | 0.32 ± 0.04 | |
| 22:1 | 0.25 ± 0.12 | 0.06 ± 0.01 | 0.1 ± 0.01 | 0.1 ± 0.05 | | 0.67 ± 0.06 | | 0.12 ± * | 0.2 ± 0.12 | 0.14 ± 0.02 | |

* Observed only in one sample

ND: Not detected

Value are means ± standard deviation
