# Supplementary material for: Metabolomic Analysis of Cold Acclimation of Arctic Mesorhizobium sp. Strain N33
Source: PLoS One. 2013 Dec 30;8(12):e84801. doi: 10.1371/journal.pone.0084801 (PMC3875568; doi:10.1371/journal.pone.0084801)
Supplement: Table S3 — Low temperature effects on the fatty acid composition of neutral lipids determined by GC-MS in arctic Mesorhizobium strain N33 (expressed as mole % of total neutral fatty acids). (DOCX) [file pone.0084801.s015.docx]

**Table S3**. Low temperature effects on the fatty acid composition of neutral lipids determined by GC-MS in arctic *Mesorhizobium* strain N33 (expressed as mole % of total neutral fatty acids).

| Experiment conditions | GT4 | | GT10 | GT21 (T0) | T1 | T2 | T3 | T4 | T5 |  |
| --- | --- | --- | --- | --- | --- | --- | --- | --- | --- | --- |
|  | **Growth at 4^o^C** | | **Growth at 10^o^C** | **Growth at 21^o^C** | **Exposed to cold temperature (4 ^o^C) for:** | | | | |  |
|  |  | |  |  | **2min** | **4min** | **8min** | **1h** | **4h** |  |
|  | | **Fatty acids from neutral lipids** | | | | | | | | |
| C12 | 0.38 ± 0.18 | | ND | 0.44 ± 0.12 | 1.41 ± 0.87 | ND | 0.53 ± 0.41 | 0.99 ± 0.24 | ND |  |
| C14 | 2.19 ± 0.96 | | 1.67 ± 0.18 | 1.33 ± 0.15 | 1.8 ± 0.31 | 1.16 ± 0.19 | 1.6 ± 0.25 | 1.29 ± 0.63 | 1.99 ± 0.13 |  |
| C14:1(11) | ND | | ND | ND | ND | ND | ND | 1.67 ± 0.51 | ND |  |
| C15 | 0.26 ± 0.14 | | 0.49 ± 0.11 | 0.32 ± 0.09 | 0.54 ± 0.05 | 0.36 ± 0.16 | 0.31 ± 0.2 | 0.38 ± 0.27 | 0.61 ± 0.07 |  |
| C16 | 32.38 ± 12.78 | | 48.32 ± 0.8 | 47.53 ± 1.16 | 54.26 ± 2.5 | 47.74 ± 9.93 | 54.37 ± 1.35 | 55.38 ± 1.34 | 52.96 ± 1.96 |  |
| C16:1(9) | 1.53 ± 0.46 | | 2.72 ± 0.62 | 1.89 ± 0.57 | 2.85 ± 0.36 | 1.37 ± 0.36 | 2.27 ± 0.37 | 2.28 ± 0.5 | 2.34 ± 0.12 |  |
| C16:1 (7) | 0.87 ± 0.75 | | 0.29 ± 0.03 | 0.27 ± 0.02 | 0.7 ± 0.51 | 0.23 ± 0.05 | 0.5 ± 0.25 | 1.05 ± 0.31 | 0.28 ± 0.02 |  |
| C18 | 26.18 ± 7.55 | | 26.34 ± 0.45 | 26.49 ± 0.59 | 28.52 ± 0.87 | 23.75 ± 2.36 | 30.01 ± 0.17 | 31.11 ± 0.38 | 30.18 ± 0.58 |  |
| C18:1(10) | 1.72 ± 1.31 | | 2.09 ± 1.74 | 3 ± 0.2 | 2.28 ± 1.04 | 1.06 ± 0.57 | 4.95 ± 0.98 | 1.61 ± 1.33 | 1.81 ± 1.98 |  |
| C18:1(9) | 28.05 ± 16.84 | | 14.5 ± 2.09 | 15.89 ± 2.13 | 5.67 ± 0.61 | 22.14 ± 10.06 | 3.23 ± 0.77 | 2.59 ± 0.6 | 6.56 ± 0.82 |  |
| C18:2(6,9) | 6.48 ± 3.59 | | 1.74 ± 0.12 | 0.28 ± 0.02 | 0.42 ± 0.14 | 0.29 ± 0.14 | 0.19 ± 0.08 | 0.27 ± 0.02 | 0.18 ± 0.02 |  |
| C18:2(9,12) | 0.24 ± 0 | | 0.43 ± 0.17 | 0.3 ± 0.07 | 0.45 ± 0.18 | 0.15 ± 0.06 | 0.55 ± 0.21 | 0.43 ± 0.05 | 0.61 ± 0.45 |  |
| C19 | 0.87 ± * | | ND | 0.08 ± 0.02 | ND | ND | ND | 0.67 ± 0.07 | 0.16 ± 0.05 |  |
| C19:1(10) | 0.51 ± * | | 0.58 ± 0.03 | 1.44 ± 0.25 | 0.87 ± 0.05 | 1.29 ± 0.36 | 0.78 ± 0.04 | 0.78 ± 0.07 | 1.56 ± 0.89 |  |
| C20 | 0.58 ± 0.02 | | 0.45 ± 0.18 | 0.43 ± 0.04 | 0.67 ± 0.06 | 0.21 ± 0.25 | 0.58 ± 0.04 | 0.59 ± 0.03 | 0.57 ± 0.04 |  |
| C20:1(11) | 0.24 ± * | | 0.28 ± 0.05 | 0.19 ± 0.06 | 0.13 ± * | 0.19 ± * | 0.18 ± 0.05 | 0.17 ± 0.06 | 0.2 ± 0.02 |  |
| C22:1(13) | 0.16 ± 0.06 | | 0.31 ± 0.31 | 0.12 ± 0.04 | 0.18 ± 0.05 | 0.27 ± 0.06 | 0.2 ± 0.01 | 0.36 ± 0.36 | 0.13 ± 0.02 |  |

*Observed only in one sample

ND- Not detected

Values are means ±standard deviation
