# Supplementary material for: Metabolomic Analysis of Cold Acclimation of Arctic Mesorhizobium sp. Strain N33
Source: PLoS One. 2013 Dec 30;8(12):e84801. doi: 10.1371/journal.pone.0084801 (PMC3875568; doi:10.1371/journal.pone.0084801)
Supplement: Table S4 — Low temperature effects on the fatty acid composition of glycolipids determined by GC-MS in arctic Mesorhizobium strain N33 (expressed as mole % of total glycolipids). (DOCX) [file pone.0084801.s016.docx]

**Table S4**. Low temperature effects on the fatty acid composition of glycolipids determined by GC-MS in arctic *Mesorhizobium*

strain N33 (expressed as mole % of total glycolipids).

|  | GT4 | GT10 | GT21 (T0) | T1 | T2 | T3 | T4 | T5 | |
| --- | --- | --- | --- | --- | --- | --- | --- | --- | --- |
| Experiment conditions | **Growth at 4^o^C** | **Growth at 10^o^C** | **Growth at 21^o^C** | **Exposed to cold temperature (4 ^o^C) for:** | | | | | |
|  |  |  |  | **2min** | **4min** | **8min** | **1h** | **4h** | |
| Fatty acids from glycolipids | | | | | | | | |  |
| C12 | 0.09 ± 0.06 | 0.13 ± 0.06 | 0.09 ± 0.02 | 0.09 ± 0.08 | 0.03 ± * | 0.08 ± 0.00 | 0.17 ± 0.06 | 0.09 ± 0.05 | |
| C14 | 1.66 ± 0.44 | 0.57 ± 0.06 | 0.39 ± 0.15 | 0.81 ± 0.59 | 0.39 ± 0.27 | 1.17 ± 0.43 | 0.99 ± 0.3 | 0.42 ± 0.1 | |
| C14:1(11) | 0.24 ± 0.2 | 0.11 ± 0.02 | 0.07 ± 0.0 | 0.13 ± * | NA | ND | 0.31 ± 0.11 | 0.05 ± 0.01 | |
| C15 | 0.32 ± 0.04 | 0.52 ± 0.06 | 0.52 ± 0.04 | 0.49 ± 0.04 | 0.48 ± 0.19 | 0.63 ± 0.05 | 0.51 ± 0.16 | 0.81 ± 0.01 | |
| C16 | 14.92 ± 7.77 | 8.02 ± 0.9 | 9.77 ± 1.03 | 15.83 ± 2.44 | 11.22 ± 3.31 | 18.51 ± 4.70 | 18.32 ± 8.29 | 10.28 ± 1.97 | |
| C16:1(9) | 1.96 ± 0.94 | 1.22 ± 0.18 | 1.22 ± 0.11 | 1.03 ± 0.09 | 1.36 ± 0.97 | 1.37 ± 0.39 | 1.11 ± 0.55 | 0.51 ± 0.18 | |
| C16:1 (7) | 0.3 ± 0.13 | 0.18 ± 0.01 | 0.17 ± 0.13 | 0.1 ± 0.02 | 0.93 ± 1.18 | 0.13 ± 0.01 | 0.26 ± 0.21 | 0.1 ± 0.09 | |
| C18 | 7.92 ± 2.83 | 4.05 ± 0.36 | 8.5 ± 0.39 | 9.42 ± 0.79 | 13.56 ± 9.95 | 10.55 ± 1.61 | 11.69 ± 2.5 | 8.35 ± 0.73 | |
| C18:1(10) | 6.64 ± * | 8.84 ± 0.17 | 9.3 ± 1.1 | 7.52 ± 1.83 | 8.2 ± 3.16 | 13.2 ± 5.95 | 12.05 ± 15.81 | 1.47 ± 0.56 | |
| C18:1(9) | 63.46 ± 7.2 | 70.71 ± 1.19 | 64.67 ± 2.7 | 60.22 ± 4.49 | 54.39 ± 25.87 | 46.42 ± 6.86 | 44.9 ± 15.54 | 61.68 ± 3.74 | |
| C18:2(6,9) | 4.78 ± 2.07 | 3.72 ± 0.2 | 0.29 ± 0.08 | 0.25 ± 0.03 | 0.59 ± 0.56 | 0.13 ± 0.04 | 0.14 ± 0.08 | 0.19 ± 0.01 | |
| C18:2(9,12) | 0.55 ± 0.58 | 0.5 ± 0.31 | 0.25 ± 0.02 | 0.25 ± 0.12 | 0.2 ± 0.04 | 0.42 ± 0.22 | 2.7 ± 2.99 | 1.13 ± 1.81 | |
| C19 | 0.81 ± 1.08 | 0.04 ± 0 | 0.05 ± 0.01 | 0.09 ± 0.03 | 0.18 ± 0.1 | 0.09 ± 0.01 | 0.11 ± 0.02 | 0.12 ± 0.02 | |
| C19:1(10) | 0.14 ± 0.06 | 0.64 ± 0.13 | 4.01 ± 0.03 | 3.1 ± 1.02 | 6.52 ± 5.73 | 6.49 ± 1.47 | 6.74 ± 1.78 | 13.68 ± 3.2 | |
| C20 | 0.07 ± 0.01 | 0.06 ± 0 | 0.29 ± 0.33 | 0.1 ± 0.01 | 0.22 ± 0.19 | 0.11 ± 0.01 | 0.11 ± 0.01 | 0.13 ± 0.05 | |
| C20:1(11) | 0.27 ± 0.07 | 0.48 ± 0.04 | 0.45 ± 0.11 | 0.39 ± 0.04 | 1.01 ± 0.91 | 0.35 ± 0.02 | 0.35 ± 0.04 | 0.46 ± 0.03 | |
| C22:1(13) | 0.45 ± 0.15 | 0.21 ± 0.05 | 0.19 ± 0.08 | 0.31 ± 0.05 | 1.27 ± 0.92 | 0.33 ± 0.05 | 0.33 ± 0.09 | 0.52 ± 0.13 | |

*: Observed only in one sample

ND: Not detected

Value are means ± standard deviation
