# Supplementary material for: Metabolomic Analysis of Cold Acclimation of Arctic Mesorhizobium sp. Strain N33
Source: PLoS One. 2013 Dec 30;8(12):e84801. doi: 10.1371/journal.pone.0084801 (PMC3875568; doi:10.1371/journal.pone.0084801)
Supplement: Table S5 — Low temperature effects on the fatty acid composition of phospholipids determined by GC-MS in arctic Mesorhizobium strain N33 (expressed as mole % of total phospholipids). (DOCX) [file pone.0084801.s017.docx]

**Table S5**. Low temperature effects on the fatty acid composition of phospholipids determined by GC-MS in arctic *Mesorhizobium* strain N33 (expressed as mole % of total phospholipids).

| Experiment conditions | GT4 | GT10 | GT21 (T0) | T1 | T2 | T3 | T4 | T5 | |
| --- | --- | --- | --- | --- | --- | --- | --- | --- | --- |
|  | **Growth at 4^o^C** | **Growth at 10^o^C** | **Growth at 21^o^C** | **Exposed to cold temperature (4 ^o^C) for** | | | | | |
|  |  |  |  | **2min** | **4min** | **8min** | **1h** | **4h** | |
| Fatty acids from phospholipids | | | | | | | | |  |
| C12 | 0.06 ± 0.02 | 0.03 ± 0.02 | ND | 0.03 ± 0.02 | ND | 0.01 ± * | 0.36 ± 0.58 | 0.04 ± 0.04 | |
| C14 | 0.19 ± 0.12 | 0.23 ± 0.24 | 0.06 ± 0.01 | 0.27 ± 0.08 | 0.04 ± 0.03 | 0.1 ± 0.06 | 0.55 ± 0.29 | 0.14 ± 0.01 | |
| C14:1(11) | 0.02 ± * | 0.03 ± 0.04 | ND | ND | ND | ND | 0.22 ± 0 | 0.04 ± 0.03 | |
| C15 | 0.28 ± 0.04 | 0.39 ± 0.09 | 0.57 ± 0.05 | 0.56 ± 0.02 | 0.4 ± 0.28 | 0.83 ± 0.11 | 0.26 ± 0.37 | 0.77 ± 0.07 | |
| C16 | 9.46 ± 1.11 | 7.17 ± 0.99 | 16.29 ± 1.13 | 13.83 ± 0.08 | 9.25 ± 6.49 | 12.63 ± 0.65 | 10.81 ± 0.31 | 11.47 ± 1.53 | |
| C16:1(9) | 0.81 ± 0.08 | 0.92 ± 0.1 | 0.86 ± 0.1 | 0.82 ± 0.03 | 0.64 ± 0.45 | 0.74 ± 0.02 | 0.75 ± 0.08 | 0.78 ± 0.18 | |
| C16:1 (7) | 0.21 ± 0.06 | 0.19 ± 0.13 | 0.09 ± 0.01 | 0.08 ± 0 | 0.06 ± 0.04 | 0.05 ± 0 | 0.11 ± 0.08 | 0.08 ± 0.02 | |
| C18 | 0.86 ± 0.79 | 0.77 ± 0.03 | 5.18 ± 0.59 | 4.06 ± 0.15 | 2.84 ± 2.03 | 5.14 ± 0.5 | 4.57 ± 0.03 | 5.33 ± 1.69 | |
| C18:1(10) | 1.17 ± 0.25 | 1.45 ± 0.47 | 1.52 ± 0.2 | 1.99 ± 0.08 | 4.47 ± 5.01 | 1.13 ± 0.09 | 1.35 ± 0.1 | 2.56 ± 3.21 | |
| C18:1(9) | 68.63 ± 1.52 | 73.53 ± 2.56 | 56.62 ± 4.65 | 64.97 ± 0.98 | 72.45 ± 11.34 | 53.53 ± 2.32 | 56.49 ± 2.38 | 47.54 ± 4.23 | |
| C18:2(6,9) | 17.39 ± 1.1 | 12.28 ± 1.7 | 0.72 ± 0.05 | 0.64 ± 0.07 | 0.55 ± 0.38 | 0.29 ± 0.02 | 0.34 ± 0.07 | 0.27 ± 0.24 | |
| C18:2(9,12) | 0.18 ± 0.06 | 0.19 ± 0.17 | 0.02 ± 0.01 | 0.08 ± 0.05 | 0.02 ± 0.02 | 0.05 ± 0.06 | 0.04 ± 0.01 | 2.01 ± 3.05 | |
| C19 | 0.13 ± 0.19 | 0.04 ± 0.01 | 0.07 ± 0 | 0.05 ± 0.01 | 0.06 ± 0.04 | 0.08 ± 0.02 | 0.11 ± 0.02 | 0.1 ± 0.03 | |
| C19:1(10) | 0.33 ± 0.02 | 2.47 ± 0.2 | 17.39 ± 2.92 | 12.13 ± 1.09 | 8.9 ± 6.56 | 24.88 ± 1.92 | 24.01 ± 2.2 | 28.41 ± 7.15 | |
| C20 | 0.02 ± 0.01 | 0.02 ± 0 | 0.04 ± 0.01 | 0.03 ± 0 | 0.02 ± 0.01 | 0.04 ± 0.01 | 0.03 ± 0 | 0.06 ± 0.03 | |
| C20:1(11) | 0.21 ± 0.02 | 0.35 ± 0.03 | 0.5 ± 0.07 | 0.35 ± 0.01 | 0.26 ± 0.18 | 0.41 ± 0.02 | 0.36 ± 0 | 0.39 ± 0.03 | |
| C22:1(13) | 0.1 ± 0.02 | 0.06 ± 0.01 | 0.08 ± 0.02 | 0.12 ± 0.02 | 0.05 ± 0.04 | 0.1 ± 0.02 | 0.09 ± 0.02 | 0.07 ± 0.02 | |

* Observed only in one sample

ND: Not detected

Value are means ± standard deviation
